# A laboratory investigation into features of morphology and physiology for their potential to predict reproductive success in male frogs

**DOI:** 10.1371/journal.pone.0241625

**Published:** 2020-11-11

**Authors:** Frances Orton, Sofie Svanholm, Erika Jansson, Ylva Carlsson, Andreas Eriksson, Tamsyn Uren Webster, Tamara McMillan, Martin Leishman, Bas Verbruggen, Theo Economou, Charles R. Tyler, Cecilia Berg

**Affiliations:** 1 School of Health and Life Sciences, University of the West of Scotland, Paisley, United Kingdom; 2 Department of Environmental Toxicology, Uppsala University, Uppsala, Sweden; 3 Swansea University, Swansea, United Kingdom; 4 College of Life and Environmental Sciences, University of Exeter, Exeter, United Kingdom; National Institute of Child Health and Human Development (NICHD), NIH, UNITED STATES

## Abstract

Amphibian populations are declining globally, however, the contribution of reduced reproduction to declines is unknown. We investigated associations between morphological (weight/snout-vent length, nuptial pad colour/size, forelimb width/size) and physiological (nuptial pad/testis histomorphology, plasma hormones, gene expression) features with reproductive success in males as measured by amplexus success and fertility rate (% eggs fertilised) in laboratory maintained *Silurana*/*Xenopus tropicalis*. We explored the robustness of these features to predict amplexus success/fertility rate by investigating these associations within a sub-set of frogs exposed to anti-androgens (flutamide (50 μg/L)/linuron (9 or 45 μg/L)). In unexposed males, nuptial pad features (size/colour/number of hooks/androgen receptor mRNA) were positively associated with amplexus success, but not with fertility rate. In exposed males, many of the associations with amplexus success differed from untreated animals (they were either reversed or absent). In the exposed males forelimb width/nuptial pad morphology were also associated with fertility rate. However, a more darkly coloured nuptial pad was positively associated with amplexus success across all groups and was indicative of androgen status. Our findings demonstrate the central role for nuptial pad morphology in reproductive success in *S*. *tropicalis*, however, the lack of concordance between unexposed/exposed frogs complicates understanding of the utility of features of nuptial pad morphology as biomarkers in wild populations. In conclusion, our work has indicated that nuptial pad and forelimb morphology have potential for development as biomarkers of reproductive health in wild anurans, however, further research is needed to establish this.

## Introduction

One of the most critical wildlife conservation issues today is the global amphibian decline. In the last comprehensive global assessment (2004), one third of amphibian species were reported as threatened with extinction and more than 40% were experiencing population declines, a level greater than for any other vertebrate taxa [1]. Furthermore, in that assessment information was available to determine population status for only two thirds of amphibian species, and therefore, it is likely that the status for amphibians is even more severe. Clearly, species producing fewer offspring over their lifespan are at increased risk of decline [2] and reduced reproduction is thought to contribute to amphibian population declines [3]. However, definitive evidence is lacking, as reproductive success has received relatively little attention compared with the impacts of adult mortality on declines, for example, due to chytridiomycosis [4]. The delineation between reduced survival and reduced reproduction is important for the development of effective mitigation strategies, particularly since reproduction occurs in the aquatic environment for the vast majority of anuran amphibians, which is distinct from the terrestrial environment they inhabit as adults [5].

Reproductive health can be measured directly, for example, by counting the number of spawn clumps *in situ* (e.g. [6]) or inferred *via* assessment of reproductive investment *ex situ* (e.g. [7]) as well as *via* analysis of tadpole hatching success [8] and/or tadpole fitness [7] *ex situ*, however, these methods do not allow the direct measurement of the reproductive success of individuals. The availability of robust measures of reproductive success are needed in order to determine the contribution of reduced reproduction to amphibian declines. In anurans, the presence of ‘intersex’ (mixed ovarian/testicular tissue) has sometimes been used to infer negative impacts on male reproductive fitness, however, evidence suggests they may not necessarily be linked (for review see: [9]). Success can be measured in a variety of ways, but since anurans employ external fertilisation, the primary determinant of reproductive success is achieving and maintaining amplexus with the female during egg laying. Reproductive success of individuals can also be increased in non-amplectant males, which nevertheless fertilise eggs through the medium of sperm competition [10]. The degree to which amplexus success and sperm competition dominate fertilisation success is related to breeding modes, which are often described as ‘explosive’ and ‘prolonged’ [5], though this is an oversimplification as in reality these modes represent a continuum. At the extreme end of this continuum for ‘explosive’ breeders, ‘breeding balls’ are observed whereby many males amplect with a single female simultaneously (e.g. *Bufo bufo*), with little influence of female choice on breeding outcomes [11] and multiple paternity is commonplace (e.g. [12]). By contrast, *Silurana tropicalis* (a member of the Pipidae family) is described as a ‘prolonged’ breeder, as is the case for many Hylidae frogs [5]. In Pipidae, the breeding mode is characterised by courtship rituals, female choice and an elaborate breeding process whereby amplectant pairs perform ‘turnovers’, allowing the amplecting male exclusive access to the eggs as they are being laid [13, 14]. Due to this breeding behaviour, it is assumed that in Pipidae frogs, the amplecting male is responsible for the fertility rate, with little or no influence of sperm competition, but this has not been empirically tested. In addition to the rapid development of *S*. *tropicalis*–reaching sexual maturity within 6 months [15]–the prolonged breeding mode of this species facilitates investigations into reproductive success which can be determined *via* behavioural observations and the fact that only one male frog is in amplexus with a female frog at any one time.

Since reproduction is primarily controlled by the androgens in male anurans [16, 17], features with potential as markers of reproductive success in males are also likely to be controlled by androgens. These include secondary sexual characteristics, such as a larger and/or darkened nuptial pads [18]–which have been identified in all amphibian families analysed to date (including Ranids, Leptodactylids, Bufonids, Pipids and Hylids: [5, 19, 20])–and larger/wider forelimbs [21], compared to females. Androgens have been shown to control the development and maintenance of nuptial pads [19], with higher levels of *ar* (androgen receptor) found in male forelimbs compared to females [22]. In addition, castration of adult *Xenopus laevis* [20] or *Rana pipiens* [23] has been shown to result in a reduced nuptial pad size, that could be restored through androgen implants. Exposure to estrogens during the larval/juvenile stages has also been shown to reduce nuptial pad size [24]. In adults, exposure to anti-androgens results in a reduction in the number of keratinised hooks on nuptial pads (*Xenopus laevis*: [25]) and the flexor carpi radialis muscle size is positively regulated by androgens [26]. The nuptial pad is thought to contribute in non-random mating scenarios for anuran amphibians as changes occur in morphology, including increase in nuptial pad size and/or number occur during breeding [18]. In addition, amplecting wild male Columbia spotted frogs (*Rana luteiventris*) have been reported to have larger nuptial pads and larger forelimbs compared to lone males [27]. In addition to features of external morphology, spermatogenesis–which is an internal physiological endpoint–has been shown to be both controlled by androgens [28] and related to reproductive success [29]. Glucocorticoids are also important for reproductive success in male anurans [17], as demonstrated in relation to calling behaviour. Lower levels of corticosterone have been found in calling (i.e. reproductively active) *versus* non-calling túngara frogs (*Physalaemus pustulosus*) [30]. In addition, artificially increasing corticosterone levels *via* injection in calling wild toads (*Bufo cognatus* and *Bufo woodhousii*) results in the cessation of calling in these toads [31].

In other aquatic wildlife species, pollution has been shown to be a major contributing factor to population declines, including for fish, fish eating birds, and alligators [32]. In amphibians, after habitat loss/degradation, pollution is estimated to be the second most important cause of amphibian species decline globally [33] and specific environmental pollutants are known to cause reproductive impairment in wild and in laboratory amphibians exposed during the larval life stage (for review see: [9]). Furthermore, the vast majority of amphibians breed in aquatic environments [5] and their larvae—generally the most vulnerable developmental life stages–are therefore often exposed to mixtures of chemical pollutants.

In this study we investigated the relationships between frog size (body weight, snout-vent length (SVL)), secondary sexual characteristics (nuptial pad colour/size/histomorphology, forelimb width/size), testicular histomorphology (spermatogenesis, tubule number), gene expression (mRNA levels of *ar* [androgen receptor] and *gr* [glucocorticoid receptor] in brain/forelimb/testis) and hormone levels (androgens/corticosterone) with reproductive success in males, as defined by males gaining amplexus with a female under competition and the fertility rate of winning frogs. We conducted these assessments within groups of unexposed animals and within groups of frogs exposed to anti-androgenic chemicals (flutamide (50 μg/L), linuron (9 or 45 μg/L)). The driving aim of this work was to investigate whether features or morphology/physiology in adult frogs could predict reproductive outcomes in those frogs, including under exposure conditions. Anti-androgenic chemicals are particularly relevant for effects on male reproductive success [34] and are commonly found to induce effects at environmentally relevant concentrations [35]. In this work we were seeking to identify features associated with male reproductive success in a laboratory model species as a first step towards the development of reliable biomarker(s) of reproductive health with potential for application to wild anurans.

## Methods

### Rearing and chemical exposure conditions

The rearing conditions for the frogs, *S*. *tropicalis*, in this study have been described previously [36]. Briefly, they were reared in aged tap water (48 hours) with 50% water change 3 times per week (range: temperature, 25.4–27.1°C; dissolved oxygen, 20.9–99.3%; pH, 7.19–8.25; unionised ammonia, 0–736 μg/L, water conductivity, 448–586 μS). For the chemical treatment groups (4 replicate tanks), larvae were exposed via the water to the pesticide linuron (9 μg/L [32 nM] or 45 μg/L [181 nM]) or to the anti-androgen flutamide (50 μg/L [181 nM]) from Nieuwkoop-Faber stage 40 (~24 hours post-hatch) throughout the larval period (~ 2 months). Upon completion of metamorphosis, chemically treated frogs were placed in clean (test substance-free) water and reared to sexual maturity (32 per tank) under flow-through conditions. At 4 months post-metamorphosis, juvenile male and female frogs were placed into separate tanks to eliminate the possibility of non-controlled breeding. See S1 and S2 Methods in S1 File for further details of animal husbandry/experimental conditions and Orton *et al*. 2018 [36].

### Analysis of reproductive success

Breeding of male *S*. *tropicalis* was assessed 6 months post-metamorphosis in competitive breeding trials, whereby 2 experimental males were placed with 1 female frog of a similar age (~1.5 years) and size (~20 grams). The females were obtained from Xenopus 1 (Dexter, USA) 2 weeks prior to the beginning of the breeding trials. During the week prior to breeding, males were weighed and individually marked with visible implant elastomer tags in the webbing of their toes (Northwest Marine Technology, USA). One day prior to the breeding trial, the selected males (1 or 2 sets of two males) and females (1 or 2 individuals) were placed in holding tanks (each set of two males in 6L tanks; all female frogs in a 15L tank) in water at a lower temperature (24 ± 1°C) to stimulate breeding conditions [37]. On the day of the breeding trial, male and female frogs were given two injections of human chorionic gonadotrophin (hCG): a priming dose (20 IU) and subsequently a boosting dose 23 hours later (100 IU). Immediately prior to the boosting injection, photographs were taken of the forelimb and nuptial pad (Nikon D70, objective AF micro Nikkon 60 mm 1:2:8D) and the frogs were weighed. Forelimb width and length were analysed with ImageJ (National Institute of Health, Bethesda, MD—USA) and nuptial pad size and colour were analysed with Adobe Photoshop CS6 for (see S1 & S2 Figs in S1 File). Following the hCG boosting injection, each set of two males was placed in a breeding tank (20 L, darkened with plastic covering, containing 3 glass petri dishes to collect the spawn), and left for 20–25 minutes before the introduction of a pre-weighed female. The order in which females were added to breeding tanks was randomised.

Two breeding trials were undertaken with each male, ensuring a similar recovery time between breeding trials (minimum of 10 days). In total 158 breeding trials were undertaken (92 trials for the first breeding, and 66 for the second breeding). The breeding trial for each tank (= each male pair+ unexposed female) was initiated at the time when the female was placed into the breeding tank with the males. After 60 minutes, and thereafter every 45 minutes, tanks were assessed to identify the individual male in amplexus and whether spawning had occurred. The identity of the successful male was determined by visual inspection; i.e. the male that was in amplexus during spawning. Six hours after the first amplexus was observed, or alternatively if the frogs were not observed in amplexus for two consecutive measured 45 min time points (90 minutes without amplexus), frogs were removed from the breeding tanks (maximum breeding trial time = 10 hours. Photographs were taken of the spawn immediately following removal of the frogs to determine the number of eggs spawned (fecundity). A sub-sample of approximately 70% of the eggs were then removed from each breeding tank by lifting 3 glass petri dishes out of the breeding tank and placing in a new 2 L tank (26°C) to assess fertilisation rate (percentage of fertilised eggs). This was assessed by comparing photographs of the eggs taken immediately after their collection from the breeding tanks and those taken 26 hours after initiation of amplexus, when the fertilised eggs could be distinguished through their development (elongation, see S3 Methods in S1 File).

### Analysis of relationships between morphological/physiological endpoints and reproductive success

For the two breeding trials in this study, different combination of endpoints were analysed: In the first breeding trial only morphological (non-destructive) endpoints were measured. In the second breeding trial both morphological and physiological endpoints (that required animal termination) were measured. Males were normally paired within treatment groups in order to investigate relationships between measured endpoints and amplexus success/fertility rate within each group ((first breeding trial = 92 trials; second breeding trial = 27 [out of 66] trials). In a sub-set of the frogs from the second breeding trial (39 out of 66 trials), unexposed males were paired with exposed males to additionally investigate the impacts of unexposed/exposed frog combination on breeding dynamics.

#### Morphological endpoints

The morphological endpoints analysed were: frog weight/SVL, nuptial pad size/colour/proportion (the proportion of the total size of the nuptial pad that was dark) and forelimb width/size (length x width). Immediately prior to the boosting injection, frogs were weighed, and photographs were taken of the forelimb and nuptial pad (Nikon D70, objective AF micro Nikkon 60 mm 1:2:8D). Photographs were analysed with ImageJ (National Institute of Health, Bethesda, MD—USA) to determine forelimb width and length and Adobe Photoshop CS6 for nuptial pad size and colour. For further details, see Orton et al. [36].

#### Physiological endpoints

Frogs were euthanised after the second breeding trial, they were weighed and their SVL measured. The frogs were then sacrificed by decapitation under anaesthesia (3% tricaine methanesulfonate, pH 7.5, 2 mins), blood was collected for hormone analyses, the left forelimb/brain/gonad were dissected and placed in RNAlater for analysis of gene expression (*gr*/*ar*/*rpl8 [ribosomal protein L8 –*housekeeping gene]) and the right testis and right forelimb were dissected and fixed in neutral buffered formalin (4%) for histological analysis. The physiological endpoints analysed were nuptial pad histomorphology (number of breeding glands, number/size serous glands, number of keratinised hooks), testis weight, testicular histomorphology (number of tubules, number spermatogonia, number spermatocytes, number germ nests, proportion of germ nests with the dominant cell type as: spermatocytes, spermatids or spermatozoa), gene expression (forelimb/brain/gonad mRNA levels of *ar*/*gr [normalised to rpl8 mRNA level]*) and plasma hormones (androgens, corticosterone).

*Testicular histomorphology*. Gonadal tissue for histological analysis was embedded in hydroxyethyl methacrylate (Technovit 7100, Heraeus Kulzer, Wehrheim, Germany), sectioned (at 2 μm) and stained with haematoxylin-eosin for analysis using light microscopy (Leitz, Laborlux 12, Leica AB, Kista, see S3 Methods in S1 File). Evaluation of testis histomorphology comprised of an analysis of germ cell types (spermatogonia, spermatocytes, spermatozoa) and testis structural features (cell nests, tubules) in 10 randomly taken pairs per treatment (20 frogs per treatment, see S4 Methods in S1 File).

*Nuptial pad histomorphology*. Forelimb tissue for histological analysis was embedded in paraffin or hydroxyethyl methacrylate (Technovit 7100, Heraeus Kulzer, Wehrheim, Germany) and samples were sectioned (5 μm) and stained with haematoxylin-eosin for analysis using light microscopy (Novex B-range). Transverse sections were cut along the entire length of the arm and three sections, at one quarter (25%), halfway (50%), and three quarters (75%) of the way through the tissue were selected for analysis. The total number of special mucous glands/breeding glands (glands containing mucous, staining pink), the number and size of normal mucous glands/serous glands (glands not containing mucous, staining pink/purple) and keratinised hooks (protruding hook features) from the 3 selected sections were counted. See S3 Fig in S1 File for histomicrographs of the features analysed.

*Gene expression analysis*. Tissue samples (brain, gonad, forelimb) were homogenized and lysed, RNA extracted/isolated, and cDNA synthesised. cDNA samples were tested in two independent PCR runs. Expression levels of genes related to reproductive behaviour were analysed (*ar* and *gr*) using RT-qPCR, and with efficiency-corrected normalisation to the *rpl8* housekeeping gene. Each sample was run in duplicate and a no-template control (NTC) sample with water instead of cDNA was included throughout. qPCR primers were optimised using standard curves and had efficiency of 85–115 and r^2^ > 0.9. Melt curve analysis was used to determine amplicon specificity and the absence of amplification in the NTCs (4 runs/plates were excluded). See S5 Methods and S1 Table in S1 File for details of primer design/sequence and PCR conditions.

*Plasma hormone analysis*. Following euthanasia, blood was collected in heparinised capillary tubes by cardiac puncture, centrifuged (5 minutes, 1000g) and the plasma withdrawn and snap frozen in liquid nitrogen. Upon thawing, plasma was extracted twice with ethyl acetate (each time with equal volumes of ethylacetate:plasma), evaporated under nitrogen gas, and resuspended in 2:1 volume ethanol:plasma ratio, for use in hormone assays (extraction efficiency: testosterone/dihydrotestosterone– 98%, corticosterone– 95%). The final dilution for assays was x200 for both corticosterone and for testosterone/dihydrotestosterone. Corticosterone was quantified using radioimmunoassay (polyclonal antibody from Abcam, cross-reactivity < 1.5%) and testosterone/dihydrotestosterone (equal binding affinity to the antibody) with enzyme-linked immunoassay (Oxford Biomedical Research, cross-reactivity < 1%). All samples were run in duplicate in each assay. The intra-assay variability for corticosterone was 9.3% (2 assays) and for dihydrotestosterone/ testosterone was 6.9% (4 assays).

### Statistics

Generalised linear regression modelling (GLM) implemented in the Bayesian framework was used to analyse output from the breeding trials, with data from the first and second breeding trials analysed separately. GLM was run to analyse the effects of morphology (weight, SVL, nuptial pad size/colour/proportion (the proportion of the total size of the nuptial pad that contained dark pigmentation), forelimb width/size (length x width)) and physiology (nuptial pad breeding gland number, serous gland number/size, keratinised hook number, forelimb/brain/gonad mRNA levels of *ar*/*gr*, plasma testosterone /corticosterone, testis weight and testis histomorphology [number of tubules, number spermatogonia, number spermatocytes, number germ nests, proportion of germ nests with the dominant cell type as spermatocytes, spermatids or spermatozoa]) on winning the competition for amplexus with the female (Binomial distribution) and for fertility rate (percentage fertilised eggs–Gamma distribution). For the amplexus models, data from both frogs in each pair (winning and losing frogs) were included as these frogs were in competition with each other. For fertility models, only data from the winning frog was included using the assumption that only the winning frog contributed to the fertility observed (see Introduction). Since characteristics of females (unexposed) had the potential to contribute to fertility, fecundity (number of eggs laid) were also included in fertility models. For all models run for second breeding trial data, the time between the two breedings (‘interval’) was included to allow for any potential confounding influence of recovery time, which ranged from 8–14 days between individual frogs. There were no effects of treatment on winning the competition (GLM, *p* > 0.17) and therefore breeding trial data from the within group and between group (unexposed *versus* exposed) conditions were combined. This was done to increase the *n* number, which was needed to be able to run the models, however, ‘treatment’–defined as belonging to linuron low, linuron high or flutamide treated groups–was included as a covariate in order to control for any non-significant subtle effects of treatment on model outcomes. Due to the large number of endpoints, in order to be able to run the models, variables were grouped for analysis within categories (e.g. nuptial pad histomorphology). See S2, S3 Tables in S1 File for details of models run, covariates included and category groupings. Effects were deemed significant if the probability of the effect coefficient being zero was less than 0.05 –equivalent to a *p*-value of 0.05, for both frogs (amplexus success models) or for the winning frog only (fertility models). Finally, a simple GLM (Gaussian) was used to investigate associations between nuptial pad colour and other measured endpoints by combining all measured endpoints from other categories (i.e., excluding nuptial pad size/proportion, which could act as confounders in the model) and then sequentially removing variables which contributed least to the data variability until the best fit for the remaining data was achieved (lowest Akaike Information Criterion [AIC] value).

### Ethics

All experiments were approved by the Uppsala Ethical Committee and carried out in accordance with relevant national guidelines and regulations.

## Results

### Breeding trials

In > 90% of the breeding trials frogs had entered into amplexus occurred by the time of the second observation (105 minutes (60 + 45 minutes)) and egg laying occurred by the time of the fourth observation (195 minutes (60 + 4 5+ 45 + 45)). In 7 out of the 148 trials there was no spawning event and these samples were excluded from the analyses. In 118 (84%) of the remaining 141 trials, only one male was observed in amplexus during spawning and this ‘winning’ male was attributed the observed fertility. For the remaining 16% (23/141) of the trials, it was unclear which male won the competition for the female and these data were also excluded from the subsequent analyses. Amplectant frogs naturally separated 6 hours after initiation of amplexus in 82% of cases. There was no effect of treatment on the amplexus success when in competition with unexposed control frogs (GLM, *p* > 0.17) and the covariate “treatment” was not associated with reproductive success (amplexus/fertility) in any models run. No associations for the covariate ‘interval’, which were included in amplexus success models for the second breeding trial, were observed. The covariate ‘egg number’ (included for all fertility models) was not associated with fertility rate in unexposed frogs but was positively associated for some of the models run in both the first and second breeding trials for linuron low/high exposed frogs (Table 1 and S11-S14 Tables in S1 File). For details of all models tested and outcomes see S2-S15 Tables in S1 File.

**Table 1:** Associations between morphology and physiology endpoints and fertility rate in *Xenopus tropicalis* in unexposed frogs and those exposed to the anti-androgenic herbicide linuron (significant associations indicated by bold and italic text–for full results see S11-S14 Tables in S1 File).

**Morphology Breeding 1**											
*Unexposed*				*Linuron Low*				*Linuron High*			
*Percentiles*	*5%*	*50%*	*95%*	*Percentiles*	*5%*	*50%*	*95%*	*Percentiles*	*5%*	*50%*	*95%*
(Intercept)	-0.42	-0.09	0.25	(Intercept)	-1.05	-0.62	-0.16	(Intercept)	-0.73	-0.38	-0.03
Egg number	-0.10	0.27	0.65	***Egg number***	***0*.*04***	***0*.*57***	***1*.*08***	***Egg number***	***0*.*14***	***0*.*51***	***0*.*89***
Weight	-0.34	0.05	0.44	Weight	-1.19	-0.52	0.15	***Weight***	***-0*.*86***	***-0*.*44***	***-0*.*03***
Forelimb Width	-0.22	0.23	0.69	***Forelimb Width***	***0*.*12***	***0*.*61***	***1*.*08***	Forelimb Width	-0.54	-0.02	0.46
Forelimb Size	-0.41	0.06	0.54	***Forelimb Size***	***0*.*01***	***0*.*60***	***1*.*18***	Forelimb Size	-0.11	0.37	0.86
**Nuptial Pad morphology Breeding 1**											
*Unexposed*				*Linuron Low*				*Linuron High*			
*Percentiles*	*5%*	*50%*	*95%*	*Percentiles*	*5%*	*50%*	*95%*	*Percentiles*	*5%*	*50%*	*95%*
(Intercept)	-0.41	-0.08	0.25	(Intercept)	-1.06	-0.64	-0.22	(Intercept)	-0.75	-0.38	0.00
Egg number	-0.10	0.27	0.65	***Egg number***	***0*.*01***	***0*.*44***	***0*.*90***	***Egg number***	***0*.*16***	***0*.*57***	***0*.*97***
Proportion	-0.30	0.04	0.40	***Proportion***	***0*.*38***	***0*.*87***	***1*.*39***	Proportion	-0.19	0.22	0.62
Colour	-0.29	0.08	0.45	Colour	-0.32	0.15	0.64	Colour	-0.06	0.30	0.68
Size	-0.25	0.14	0.54	Size	-0.59	-0.15	0.30	Size	-0.26	0.13	0.54
**Nuptial Pad Morphology Breeding 2**											
*Unexposed*				*Linuron Low*				*Linuron High*			
*Percentiles*	*5%*	*50%*	*95%*	*Percentiles*	*5%*	*50%*	*95%*	*Percentiles*	*5%*	*50%*	*95%*
(Intercept)	-0.92	-0.37	0.18	(Intercept)	-1.05	-0.59	-0.10	(Intercept)	-0.29	0.05	0.38
Egg number	-0.89	-0.01	0.93	***Egg number***	***0*.*10***	***0*.*64***	***1*.*20***	Egg number	-0.09	0.31	0.73
Interval	-1.02	-0.25	0.44	Interval	-0.04	0.51	1.03	Interval	-0.36	0.17	0.66
Proportion	-0.98	-0.25	0.48	Proportion	-0.36	0.28	0.91	***Proportion***	***0*.*17***	***0*.*63***	***1*.*12***
Colour	-0.81	0.03	0.84	Colour	-0.24	0.43	1.10	Colour	-0.69	-0.24	0.19
Size	-0.36	0.25	0.91	Size	-1.21	-0.46	0.30	Size	-0.99	-0.44	0.14
**Androgens and Androgen Receptor Breeding 2**											
*Unexposed*				*Linuron Low*				*Linuron High*			
*Percentiles*	*5%*	*50%*	*95%*	*Percentiles*	*5%*	*50%*	*95%*	*Percentiles*	*5%*	*50%*	*95%*
(Intercept)	-1.02	-0.48	0.11	(Intercept)	-0.93	-0.48	-0.04	(Intercept)	-0.26	0.10	0.48
Egg number	-0.60	0.12	0.83	***Egg number***	***0*.*16***	***0*.*63***	***1*.*15***	Egg number	-0.31	0.07	0.45
Interval	-0.72	-0.11	0.50	Interval	-0.40	0.16	0.76	Interval	-0.73	-0.27	0.17
Testosterone	-0.39	0.21	0.86	Testosterone	-0.50	0.12	0.75	Testosterone	-0.22	0.18	0.60
Testis ar*	-1.16	-0.44	0.14	Testis ar*	-0.31	0.22	0.76	Testis ar*	-0.52	-0.12	0.30
Brain ar*	-0.46	0.15	0.78	Brain ar*	-1.31	-0.61	0.00	Brain ar*	***0*.*02***	***0*.*42***	***0*.*84***
Forelimb ar*	-2.32	0.16	2.54	Forelimb ar*	-0.60	0.45	1.44	Forelimb ar*	-1.05	-0.35	0.31
**Corticosterone and Glucocorticoid Receptor Breeding 2**											
*Unexposed*				*Linuron Low*				*Linuron High*			
*Percentiles*	*5%*	*50%*	*95%*	*Percentiles*	*5%*	*50%*	*95%*	*Percentiles*	*5%*	*50%*	*95%*
(Intercept)	-1.00	-0.41	0.18	(Intercept)	-1.00	-0.50	0.01	(Intercept)	-0.29	0.08	0.47
Egg number	-1.00	-0.09	0.84	Egg number	-0.03	0.55	1.18	Egg number	-0.31	0.08	0.46
Interval	-0.68	-0.05	0.55	Interval	-0.42	0.19	0.86	Interval	-0.68	-0.26	0.17
Forelimb gr*	-0.83	0.13	1.04	Forelimb gr*	-0.77	-0.14	0.47	Forelimb gr*	-0.59	-0.14	0.29
Brain gr*	-0.66	0.13	0.94	Brain gr*	-0.90	-0.33	0.24	Brain gr*	***0*.*25***	***0*.*63***	***1*.*06***
Testis gr*	-1.15	-0.39	0.30	Testis gr*	-0.26	0.32	0.93	Testis gr*	-0.35	0.08	0.52
Corticosterone	-0.78	-0.03	0.63	Corticosterone	-0.70	-0.01	0.59	Corticosterone	-0.33	0.15	0.68

* Gene expression (mRNA levels) normalised to levels of housekeeping gene *rpl8*.

### Relationships between morphology and reproductive success

#### Unexposed frogs

**Fig 1:**
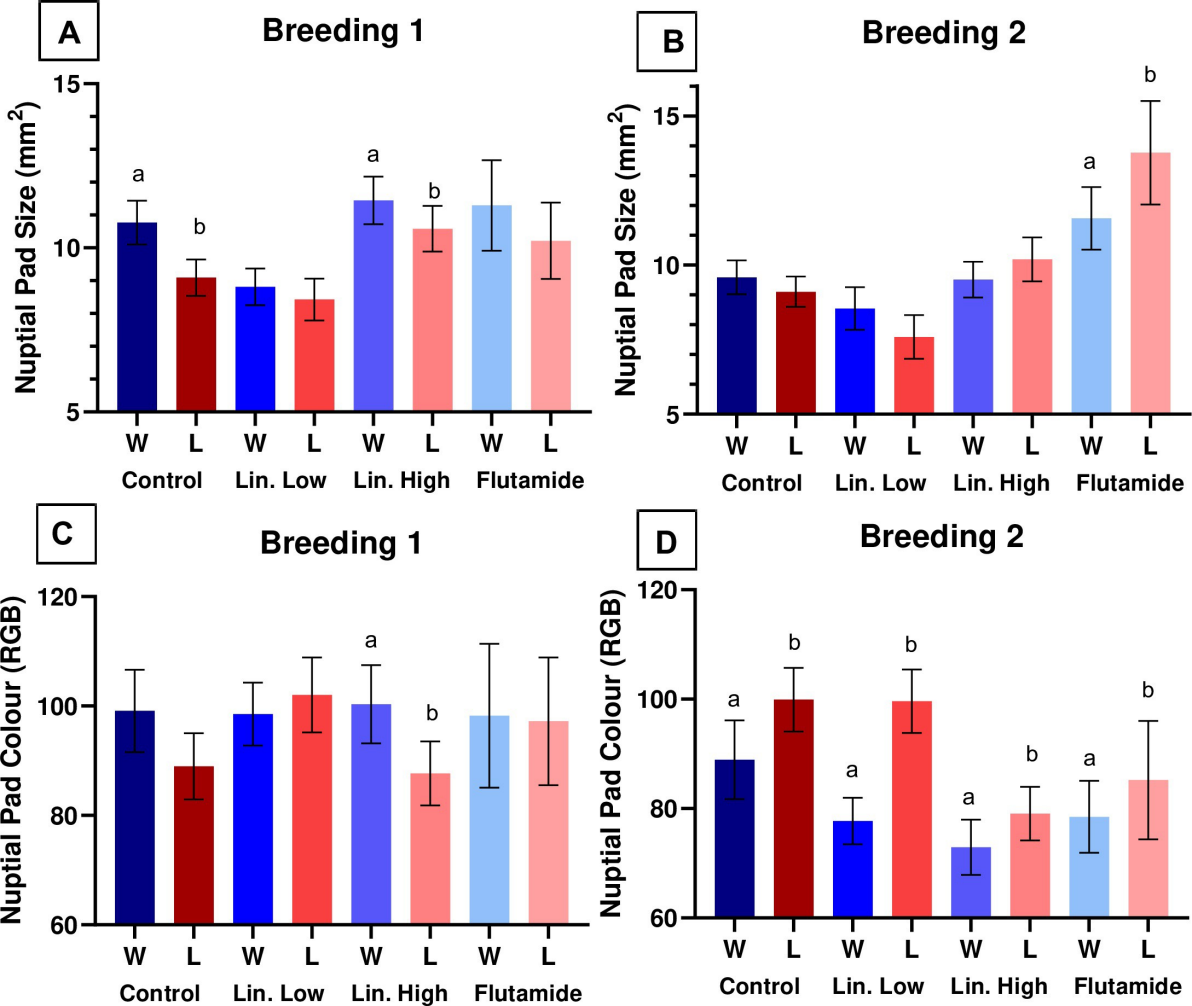
Morphological features that were significantly associated with amplexus success (winning the competition for the female) in the first and second breeding trials. Data analysed by GLM (Binomial distribution). Nuptial pad size in the first (A) and second (B) breeding trials, and nuptial pad colour (darker colour = lower number) in the first (C) and second (D) breeding trials in winning frogs (W = blue bars) and in losing frogs (L = red/pink bars). ‘a’ & ‘b’ signify significant differences (equivalent to *p* < 0.05: probability of the regression coefficient being zero is smaller than 5%) between winning/losing frogs for each treatment group (Control, Lin. (Linuron) Low, Lin. High, Flutamide). Values shown are mean ± SE.

In the first breeding trial, nuptial pad size was positively associated with amplexus success (Fig 1A), whereas in the second breeding trial, a more darkly coloured nuptial pad (lower number) was positively associated with amplexus success (Fig 1D). Neither weight, SVL nor forelimb width/size were associated with amplexus success (S5 & S6 Tables in S1 File) and there were no correlations of any measured endpoints with fertility rate (S11-S14 Tables in S1 File).

#### Exposed frogs

**Fig 2:**
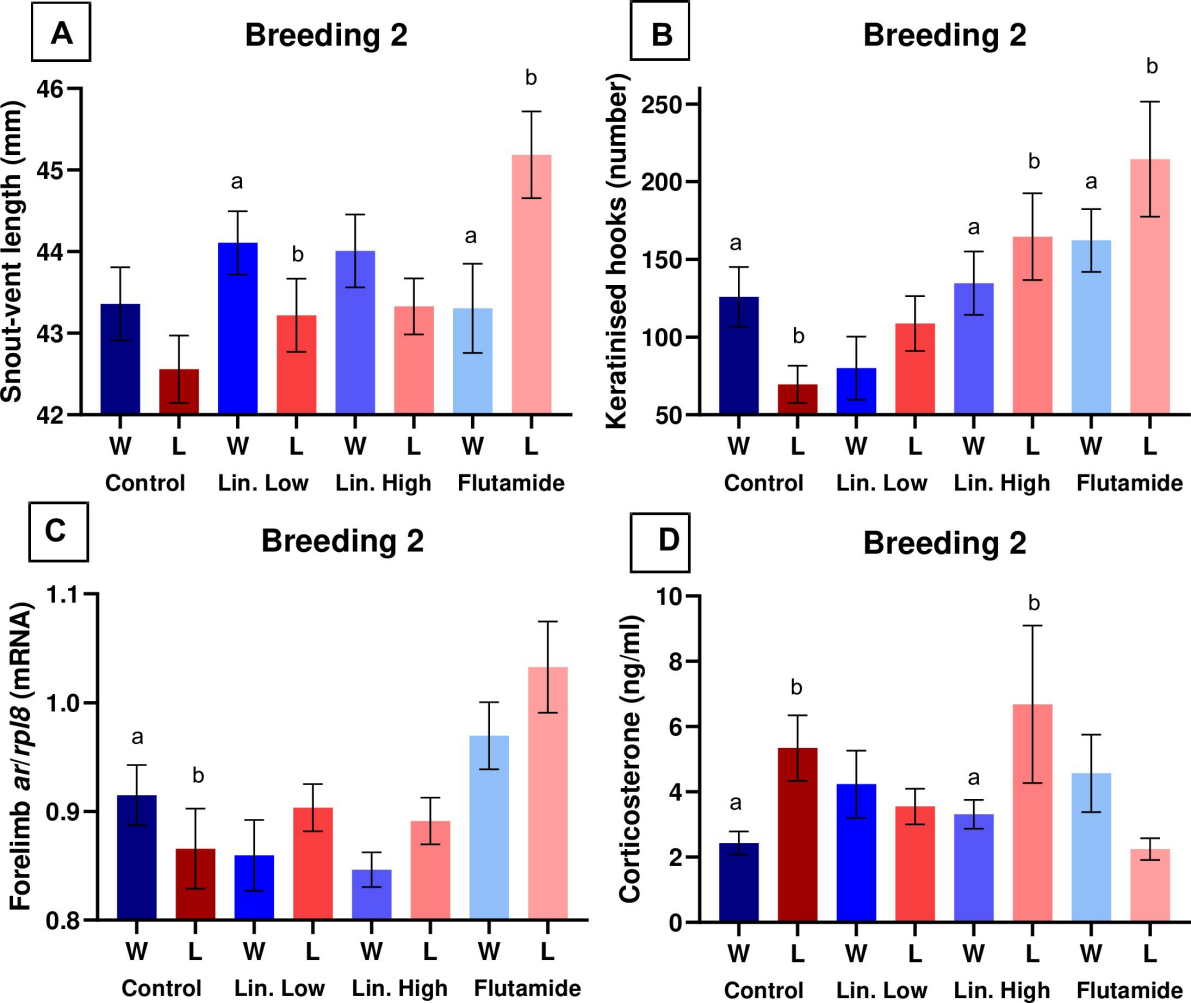
Morphological/physiological features that were significantly associated with amplexus success (winning the competition for the female) in the second breeding trial. Data analysed by GLM (Binomial distribution), models included the co-variates “interval”, representing the time between the first and second breeding trials, and “treatment”, representing the different treatment groups. Snout-vent length (A), the number of keratinised hooks (B), forelimb *ar*/*rspl8* mRNA (C) and corticosterone (D) in winning frogs (W = blue bars) and in losing frogs (L = red/pink bars). ‘a’ & ‘b’ signify significant differences (equivalent to *p* < 0.05: probability of the regression coefficient being zero is smaller than 5%) between winning/losing frogs for each treatment group (Control, Lin. (Linuron) Low, Lin. High, Flutamide). Values shown are mean ± SE.

In the first breeding trial, nuptial pad size and a more lightly coloured nuptial pad were positively associated with amplexus success (linuron high; Fig 1A and 1C). In the second breeding trial, nuptial pad size was negatively associated with amplexus success (flutamide; Fig 1B) and a more darkly coloured nuptial pad (lower number) was positively associated with amplexus success (Fig 1D). In addition, SVL (measured in second breeding trial only) was both positively (linuron low) and negatively (flutamide) associated with amplexus success (Fig 2A).

In the first breeding trial, fertility rate was negatively associated with weight (linuron high), positively associated with forelimb width/size (linuron low) and positively associated with nuptial pad proportion (Table 1 and S7 Table in S1 File). In the second breeding trial, fertility was positively associated with nuptial pad proportion and brain *ar*/*gr* (linuron high; Table 1, S14 Table in S1 File). No associations between fertility rate and measured endpoints were observed in the flutamide treated group (S11-S14 Tables in S1 File).

### Relationships between physiology and reproductive success (second breeding trial)

#### Unexposed frogs

The number of keratinised hooks (Fig 2B) and forelimb *ar* (Fig 2C) were both positively associated with amplexus success. Plasma levels of corticosterone were negatively associated with amplexus success (Fig 2D) and no correlations were observed for fertility rate with any of the measured endpoints (S11-S14 Tables in S1 File).

#### Exposed frogs

The number of keratinised hooks was negatively associated with amplexus (linuron high/flutamide; Fig 2B), as were corticosterone levels (linuron high; Fig 2D). The number of testicular tubules and the number of spermatogonia were positively associated with amplexus success (linuron low; S6 Table in S1 File), whereas brain *ar* (linuron low) and testis *ar* (linuron high) were negatively associated with amplexus success (S6 Table in S1 File). Fertility rate was positively associated with brain *ar*/*gr* in the linuron high treated group (Table 1) but no other associations were observed with fertility in the second breeding trial (S11-S14 Tables in S1 File).

## Discussion

In this study we set out to investigate the relationships between features of external morphology and physiology in male *S*. *tropicalis* with reproductive success, measured in terms of achieving amplexus and fertility rate. We investigated these relationships in unexposed frogs and in frogs that had been exposed to anti-androgenic chemicals during larval life stages. Nuptial pad morphology and physiology were associated with amplexus success in unexposed and exposed frogs, but there was little concordance in the associations observed between these groups. Nuptial pad colour was associated with amplexus success across all groups and was also associated with other androgen dependent endpoints.

Much research has established that androgens are elevated in reproductively active wild male amphibians [16] and here we show that, in unexposed males, the androgen sensitive nuptial pad was positively associated with amplexus success (i.e. size/colour of the nuptial pad, number of keratinised hooks in the nuptial pads, mRNA levels of *ar* in the forelimb). The positive relationships between these endpoints and amplexus success are not surprising, albeit we could find only one previous study in frogs showing a link between these features and reproductive success. Specifically, this was the case where wild male Columbia spotted frogs (*Rana luteiventris*) in amplexus were shown to have larger nuptial pads than unpaired males [27]. Although features of the nuptial pad were positively associated with amplexus success, androgen levels were not, i.e., plasma androgen levels in male frogs achieving amplexus did not differ from frogs that did not. Testosterone levels in male anurans have been shown to decline rapidly after mating onset [38] and the lack of correlation between plasma testosterone level and amplexus success in our study is likely due to the time lag of approximately 6 hours between the initiation of amplexus and the blood sampling. This was the case because the blood sampling method we used was destructive and could only be applied at the end of the breeding trial. We also found that lower corticosterone was associated with amplexus success, which was expected (see ‘Introduction’). Our findings support the previously suggested roles of the androgen and glucocorticoid systems as central to reproductive success in anuran amphibians [16, 17]. Body size is known to be important for achieving amplexus in some anurans [11], however, we did not observe this in our study, potentially due to the low variability in size between laboratory reared individuals.

**Fig 3:**
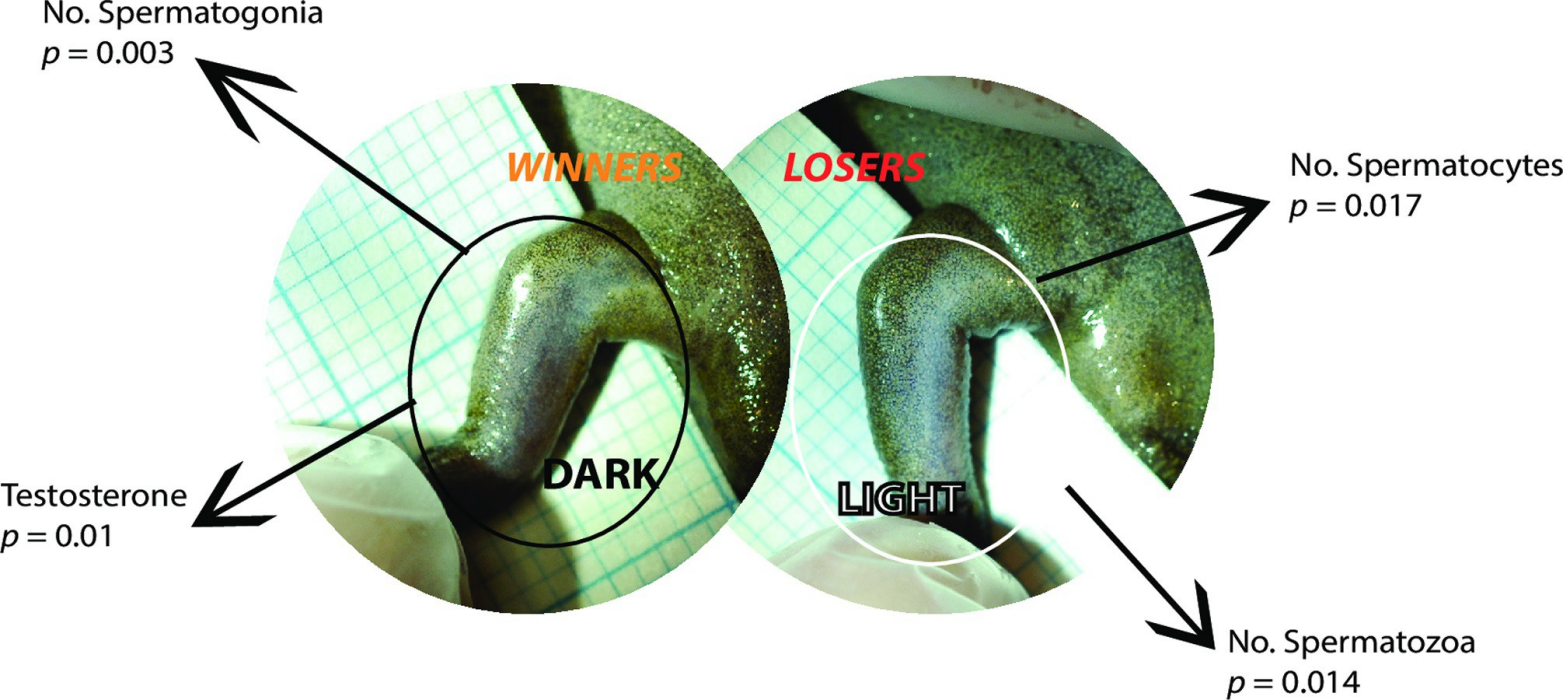
Synopsis of correlations between nuptial pad colour and reproductive physiology in *S*. *tropicalis* males using GLM (Gaussian) for all males from the second breeding trial (*n* = 63). Morphological/physiological traits were removed sequentially until the lowest AIC value was achieved (see S9 Table in S1 File for details of model).

Across all experimental groups in the second breeding trial, a darker nuptial pad in males was associated with amplexus success. This was the only measured endpoint found to be predictive achieving amplexus in both unexposed and exposed males and aligns with anecdotal evidence that frogs with a darker nuptial pad are better breeders [37]. Indeed, many of the other variables that were associated with amplexus success in the exposed groups were counterintuitive based on current understanding of amphibian reproductive biology (e.g. smaller nuptial pads, fewer keratinised hooks, lower levels of testis *ar*, lower SVL). This was surprising and we cannot currently hypothesise possible explanations for these findings since to the authors’ knowledge, this is the first study to investigate associations between morphology/physiology and amplexus success in laboratory exposed anurans. For the darkened nuptial pad, a plausible explanation for why we only observed this in the second breeding trial could be that the forelimb tissue had become sensitised because of the injection of hCG to induce breeding in the first trial leading to darkened nuptial pads in the second breeding trial. Although the mechanism driving nuptial pad colouration is not known, and proposed sensitisation is a hypothesis only, it is borne out by the fact that nuptial pads in the breeding males were overall significantly darker in the second breeding trial compared to the first breeding trial (*t-*test, *p* = 0.007). As this feature was consistent in predicting amplexus success in male frogs in the second breeding trial and thus with potential as a biomarker of reproductive success in non-naïve wild male frogs, we further investigated whether nuptial pad colour was associated with other measured endpoints (pooled data from all frogs). We found that a darker nuptial pad was associated with greater numbers of spermatogonia in the testis and higher plasma androgen levels, whereas a lighter nuptial pad was associated with more spermatocytes and more spermatozoa (Fig 3). Since frogs with a lighter nuptial pad were less likely to achieve amplexus and thus to breed with the females, we would expect these frogs to contain more spermatozoa. In addition, since androgen administration in anurans block the progress of spermatogonia to spermatocytes, resulting in higher levels of spermatogonia [28], we might also expect frogs with a darker nuptial pad to have higher levels of spermatogonia. These findings indicate that nuptial pad colour conveys information on androgen status, which would be expected given the known sensitivity of nuptial pads to androgens (see Introduction).

At first observation, our findings also suggest that nuptial pad colour has potential as a biomarker of reproductive success since it was associated with achieving amplexus across all groups. However, to be able to relate this biomarker to reproductive success, and thereby to gain understanding into the contribution of reproductive health to population declines, one would first need to determine the relationship between nuptial pad colour and fertility in the species being examined. In addition, Orton et al. [36] reported that the same frogs analysed here from the linuron high exposure group had darker nuptial pads compared to controls, contradicting its utility for making comparisons between wild populations, which are often exposed to chemicals. From this work we evidence that nuptial pad colour has the potential for development as a biomarker of reproductive success in male frogs, and in particular for assessing androgenic status in individual frogs. How nuptial pad colour might be applied to wild populations, however, is unclear as the response to polluted conditions (here anti androgens) was not as predicted. In wild anurans, nuptial pad size has been reported to be both reduced *(Engystomops pustulosus*: [39]) and increased (*Epidalea calamita*: [40]) in populations from polluted *versus* reference sites, but to the authors’ knowledge nuptial pad colour has not been measured in wild populations.

None of the measured morphological/physiological features were associated with fertility rate in unexposed frogs, indicating that amongst the winning frogs, measured endpoints did not influence fertility rate. To the authors’ knowledge, there are no previous studies that have specifically investigated relationships between morphology/physiology and fertility in wild or laboratory amphibians, though reduced spermatozoa was found in parallel with reduced fertility in *S*. *tropicalis* exposed to ethinylestradiol in the laboratory [29]. In the absence of supporting data, we can only speculate upon the potential reasons for the lack of correlations between morphology/physiology with fertility rate. They include, low variability amongst these endpoints in the winning frog group (compared to ‘amplexus success’, which included measurements from both the winning and losing frogs) and achieving amplexus acting as a pre-selection for fertility rate, whereby frogs with higher fertility achieved amplexus at the expense of frogs with lower fertility. Perhaps due to the high degree of female choice that occurs in pipid frogs [13, 14], we may expect frogs that achieve amplexus to have higher fertility rate, however, since we did not investigate fertility rate in frogs that did not achieve amplexus, we cannot assess whether this was indeed the case. Interestingly, in contrast to unexposed frogs, fertility rate was positively associated with several measured features in frogs exposed to linuron, at either low (breeding 1: forelimb width/size, nuptial pad proportion) or high (breeding 2: brain *ar*/*gr* mRNA levels) concentration exposures. This indicates that these features may have potential as biomarkers of fertility rate in wild anurans exposed to pollutants, particularly for forelimb/nuptial pad morphology, which could be applied in vulnerable populations since they can be measured non-destructively. However, similarly to the conclusions related to nuptial pad colour, at the present time the utility, or not, of these features are not known.

The findings from this study demonstrate some of the challenges of developing a biomarker of reproductive success in anurans. There are clear indications that androgen sensitive features are linked with individual male reproductive success. However, at present it is not possible to ascertain how these markers could be used for field studies where the influence of pollutants is either a likely causative factor in declines or for which levels are unknown. It would be useful to analyse for correlations between nuptial pad colour/forelimb width with fertility rate in amplectant and lone males in wild anuran populations in unpolluted/polluted regions to gain more clarity on the functional relationships between these factors and how they are altered by pollutants. Since these features can be measured non-destructively, these investigations could also be carried out in threatened/declining populations. Other useful developments for better establishing links between the role of androgens in fertility rate of wild frogs include the application of urine collection methods for measuring androgen levels non-destructively [38]. We suggest that species employing a prolonged breeding mode might be best suited to this purpose in the first instance. Finally, the frogs in this study were injected with hCG to stimulate breeding, and at present it is unknown if the correlations observed here would be present in frogs breeding under natural conditions. In conclusion, our study provides a first step towards seeking to understand whether biomarkers of reproductive success in anurans have potential to aid in the protection of declining anuran populations.

## Supporting information

S1 File (PDF)

## References

[1] Stuart SN, Chanson JS, Cox NA, Young BE, Rodrigues ASL, Fischman DL, et al Status and trends of amphibian declines and extinctions worldwide. Science. 2004;306(5702):1783–6. 10.1126/science.1103538 15486254

[2] Owens IPF, Bennett PM. Ecological basis of extinction risk in birds: Habitat loss versus human persecution and introduced predators. Proceedings of the National Academy of Sciences. 2000;97(22):12144–8. 10.1073/pnas.200223397 11005835 PMC17308

[3] Sodhi NS, Bickford D, Diesmos AC, Lee TM, Koh LP, Brook BW, et al Measuring the Meltdown: Drivers of Global Amphibian Extinction and Decline. PLoS ONE. 2008;3(2):e1636 10.1371/journal.pone.0001636 18286193 PMC2238793

[4] Skerratt LF, Berger L, Speare R, Cashins S, McDonald KR, Phillott AD, et al Spread of chytridiomycosis has caused the rapid global decline and extinction of frogs. EcoHealth. 2007;4(2):125–34.

[5] Duellman WE, Trueb L. Biology of Amphibians. Baltimore and London: John Hopkins University Press; 1994 670 p.

[6] Loman J. Rana temporaria metamorph production and population dynamics in the field–Effects of tadpole density, predation and pond drying. Journal for Nature Conservation. 2002;10(2):95–107.

[7] Bókony V, Üveges B, Ujhegyi N, Verebélyi V, Nemesházi E, Csíkvári O, et al Endocrine disruptors in breeding ponds and reproductive health of toads in agricultural, urban and natural landscapes. Science of The Total Environment. 2018;634:1335–45. 10.1016/j.scitotenv.2018.03.363 29710633

[8] Orton F, Routledge E. Agricultural intensity in ovo affects growth, metamorphic development and sexual differentiation in the Common toad (Bufo bufo). Ecotoxicology. 2011;20(4):901–11. 10.1007/s10646-011-0658-5 21448622

[9] Orton F, Tyler CR. Do hormone-modulating chemicals impact on reproduction and development of wild amphibians? Biological Reviews. 2015;90(4):1100–17. 10.1111/brv.12147 25335651

[10] Roberts JD, Byrne PG. Chapter 1—Polyandry, Sperm Competition, and the Evolution of Anuran Amphibians In: Brockmann HJ, Roper TJ, Naguib M, Mitani JC, Simmons LW, editors. Advances in the Study of Behavior. 43: Academic Press; 2011 p. 1–53.

[11] Arak A. Male-male competition and mate choice in anuran amphibians In: Bateson P, editor. Mate Choice. Cambridge: Cambridge University Press; 1983 p. 181–210. 10.1002/jat.2550030102

[12] Sztatecsny M, Jehle R, Burke T, Hödl W. Female polyandry under male harassment: the case of the common toad (Bufo bufo). Journal of Zoology. 2006;270(3):517–22.

[13] Measey GJ, Tinsley RC. Mating Behavior of Xenopus wittei (Anura: Pipidae). Copeia. 1997;1997(3):601–9.

[14] Swisher JE. Spawning turnovers in *Xenopus tropicalis*. American Zoologist. 1969;9:573–85.

[15] Berg C, Gyllenhammar I, Kvarnryd M. *Xenopus tropicalis* as a Test System for Developmental and Reproductive Toxicity. Journal of Toxicology and Environmental Health, Part A. 2009;72(3–4):219–25. 10.1080/15287390802539079 19184736

[16] Rastogi RK, Pinelli C, Polese G, D'Aniello B, Chieffi-Baccari G. Chapter 9—Hormones and Reproductive Cycles in Anuran Amphibians In: Norris DO, Lopez KH, editors. Hormones and Reproduction of Vertebrates. London: Academic Press; 2011 p. 171–86.

[17] Carr JA. Chapter 6—Stress and Reproduction in Amphibians In: Norris DO, Lopez KH, editors. Hormones and Reproduction of Vertebrates. London: Academic Press; 2011 p. 99–116.

[18] Brizzi R, Delfino G, Jantra S. An Overview of the Breeding Glands In: Jamieson BGM, editor. Reproductive Biology and Phylogeny: Science Publishers Inc; 2003 p. 253–317.

[19] Epstein MS, Blackburn DG. Histology and histochemistry of androgen-stimulated nuptial pads in the leopard frog, *Rana pipiens*, with notes on nuptial gland evolution. Can J Zool. 1997;75(3):472–7.

[20] Thomas EO, Tsang L, Licht P. Comparative Histochemistry of the Sexually Dimorphic Skin Glands of Anuran Amphibians. Copeia. 1993;1993(1):133–43.

[21] Herrel A, Gonwouo LN, Fokam EB, Ngundu WI, Bonneaud C. Intersexual differences in body shape and locomotor performance in the aquatic frog, *Xenopus tropicalis*. Journal of Zoology. 2012;287(4):311–6.

[22] Emerson SB, Greig A, Carroll L, Prins GS. Androgen Receptors in Two Androgen-Mediated, Sexually Dimorphic Characters of Frogs. General and Comparative Endocrinology. 1999;114(2):173–80. 10.1006/gcen.1999.7251 10208766

[23] Lynch LC, Blackburn DG. Effects of testosterone administration and gonadectomy on nuptial pad morphology in overwintering male leopard frogs, Rana pipiens. Amphibia-Reptilia. 1995;16(2):113–21.

[24] Iguchi T. Amphibian development, growth and reproduction assay using Xenopus tropicalis and in vitro extrogen receptor transactivation assay. ISAREN: 7th International Symposium on Amphibian and Reptilian Endocrinology and Neurobiology Universtiy of Michigan: Frontiers in Endocrinology; 2011.

[25] Wyk JHv, Pool EJ, Leslie AJ. The Effects of Anti-Androgenic and Estrogenic Disrupting Contaminants on Breeding Gland (Nuptial Pad) Morphology, Plasma Testosterone Levels, and Plasma Vitellogenin Levels in Male Xenopus laevis (African Clawed Frog). Archives of Environmental Contamination and Toxicolology 2003;44(2):0247–56. 10.1007/s00244-002-1161-z 12520397

[26] Muller ERA, Galavazi G, Szirmai JA. Effect of castration and testosterone treatment on fiber width of the flexor carpi radialis muscle in the male frog (Rana temporaria L.). General and Comparative Endocrinology. 1969;13(2):275–84. 10.1016/0016-6480(69)90249-4 4933597

[27] Greene AE, Funk WC. Sexual Selection on Morphology in an Explosive Breeding Amphibian, the Columbia Spotted Frog (Rana luteiventris). Journal of Herpetology. 2009;43(2):244–51.

[28] Rastogi RK. Seasonal cycle in anuran (Amphibia) testis: The endocrine and environmental controls. Italian Journal of Zoology. 1976;43(1–2):151–72.

[29] Gyllenhammar I, Holm L, Eklund R, Berg C. Reproductive toxicity in *Xenopus tropicalis* after developmental exposure to environmental concentrations of ethynylestradiol. Aquatic Toxicology. 2009;91(2):171–8. 10.1016/j.aquatox.2008.06.019 18692912

[30] Marler CA, Ryan MJ. Energetic constraints and steroid hormone correlates of male calling behaviour in the túngara frog. Journal of Zoology. 1996;240(3):397–409.

[31] Leary CJ, Garcia AM, Knapp R. Elevated corticosterone levels elicit non-calling mating tactics in male toads independently of changes in circulating androgens. Hormones and Behavior. 2006;49(4):425–32. 10.1016/j.yhbeh.2005.09.004 16256990

[32] Bernanke J, Köhler H-R. The Impact of Environmental Chemicals on Wildlife Vertebrates In: Whitacre D, editor. Reviews of Environmental Contamination and Toxicology. Reviews of Environmental Contamination and Toxicology. 198: Springer New York; 2009 p. 1–47. 10.1007/978-0-387-09647-6_1 19253040

[33] Díaz SJ, Settele ES, Brondízio E.S., Ngo HT, Guèze M, Agard J, et al IPBES: Global assessment report on biodiversity and ecosystem services of the Intergovernmental Science-Policy Platform on Biodiversity and Ecosystem Services Status and Trends—Drivers of change. Bonn, Germany; 2019.

[34] Kanda R. Reproductive Impact of Environmental Chemicals on Animals In: Comizzoli P, Brown JL, Holt WV, editors. Reproductive Sciences in Animal Conservation. Cham: Springer International Publishing; 2019 p. 41–70. 10.1007/978-3-030-23633-5_3 31471794

[35] Orton F, Rosivatz E, Scholze M, Kortenkamp A. Widely used pesticides with previously unknown endocrine activity revealed as in vitro anti-androgens. Environ Health Perspect. 2011;119:794–800. 10.1289/ehp.1002895 21310686 PMC3114813

[36] Orton F, Säfholm M, Jansson E, Carlsson Y, Eriksson A, Fick J, et al Exposure to an anti-androgenic herbicide negatively impacts reproductive physiology and fertility in Xenopus tropicalis. Scientific Reports. 2018;8(1):9124 10.1038/s41598-018-27161-2 29904069 PMC6002408

[37] Harland R. Harland Xenopus tropicalis Site Berkeley, California, USA, 2003.

[38] Narayan EJ, Cockrem J, Hero J-M. Changes in serum and urinary corticosterone and testosterone during short-term capture and handling in the cane toad (Rhinella marina). General and Comparative Endocrinology. 2013;191(0):225–30. 23851041 10.1016/j.ygcen.2013.06.018

[39] Orton F, Mangan S, Newton L, Marianes A. Reduced size, impaired reproductive health and altered breeding dynamics in túngara frogs (Engystomops pustulosus) from agricultural, but not suburban, ponds. Ecotoxicology. 2020; Accepted.

[40] Zamora-Camacho FJ, Comas M. Greater reproductive investment, but shorter lifespan, in agrosystem than in natural-habitat toads. PeerJ. 2017;5:e3791 10.7717/peerj.3791 28924505 PMC5600172

