# Supplementary material for: A laboratory investigation into features of morphology and physiology for their potential to predict reproductive success in male frogs
Source: PLoS One. 2020 Nov 11;15(11):e0241625. doi: 10.1371/journal.pone.0241625 (PMC7657548; doi:10.1371/journal.pone.0241625)
Supplement: S1 File — (PDF) [file pone.0241625.s001.pdf]

A laboratory investigation into features of morphology and physiology for their potential ro predict reproductive success in male frogs.

Orton F*[1], Svanholm, S[2], Janson E[2], Carlsson Y[2], Eriksson A[2], Uren Webster T[3], McMillan T[1], Leishman M[1], Verbruggen B[4], Economou T[4], Tyler CR[b4] and Berg C[b2].

1. University of the West of Scotland, UK; 2. Department of Environmental Toxicology, Uppsala University, Sweden; 3. Swansea University, UK; 4. College of Life and Environmental Sciences, University of Exeter, UK.

[b]Senior authors

* corresponding author: frances.orton@uws.ac.uk

Supplementary methods S1 – Animal husbandry

Adult male and female *X.tropicalis* were obtained from Xenopus 1 (Dexter, USA) and housed in glass tanks in a flow-through system (12:12 light:dark cycle, 26 ± 1 °C, conductivity 505 ± 20 µS/cm) for approximately two years prior to this study. Four pairs of frogs were induced to mate using human chorionic gonadotropin (hCG) as previously described[1]. Thirty-eight tadpoles (18 hours post-fertilisation at 22^{0}C, stage 40) were selected from the 2 pairs of frogs with highest fertilisation rates (estimated, > 50%) and placed in experimental tanks (15 L, *n* = 76 tadpoles per tank). Tadpoles were initially fed Sera micron and fish flakes (Sera vipan baby, Sera, Heinsberg, Germany) until forelimbs could be observed, when their food was supplemented with Frog & Tadpole bites (HBH Pet Products, Springville USA). Metamorphs were fed Frog and Tadpole bites, gradually replaced by fish pellets during 1-3 month post-metamorphosis (Tropical Excel Color, Aquatic Nature, Roeselare, Belgium).

1 Pettersson, I., Arukwe, A., Lundstedt-Enkel, K., Mortensen, A. S. & Berg, C. Persistent sex-reversal and oviducal agenesis in adult Xenopus (Silurana) tropicalis frogs following larval exposure to the environmental pollutant ethynylestradiol. *Aquatic Toxicology* **79**, 356-365 (2006).

Supplementary methods S2 – Rearing conditions

Tadpoles were exposed to nominal concentrations of linuron ('low' - 32 nM/9 µg/L or 'high' – 181 nM/45 µg/L) or flutamide (181 nM/50 µg/L: purity > 98 %, Sigma Aldrich, USA) in acetone (0.0008%) or to acetone only (0.0008%: hereaf er referred to as 'controls') using a semi-static system (50% water change, x3 per week) to maintain good water quality[1]. Aquatic half life for flutamide has been reported to be 49 days[2], though equivalent information for flutamide was not available. Flutamide and linuron levels in the control and exposure water were measured using gas chromatography/mass spectroscopy (see table S2). Triplicate tanks were used for each experimental group (total = 12 tanks) and the placing of tanks in the experimental room was randomised. Tanks were placed in water channels to maintain constant temperature, and this was checked daily (26 ± 1 °C), together with conductivity of water used for water changes (505 ± 20 µS/cm). The photoperiod cycle was 12:12 light:dark. Tank water temperature, dissolved oxygen, pH and nitrite/ammonia levels were measured weekly. Flutamide and linuron concentrations were analysed using GCMS before water changes and immediately af er water changes. For flutamide, analysis of the tank water concentrations were done bi-weekly in flutamide and control tanks (5 before + 5 af er, see:[3]) and for linuron, analysis of tank water concentrations were done every three weeks in linuron and control tanks (3 before + 1 af er, see:[4]). The chemical analyses were undertaken at Umea University (flutamide) and at the Swedish University of agricultural sciences (linuron). At completion of metamorphosis (NF stage 66), exposure ceased and individuals were placed in new tanks containing test substance-free water (32 per tank, water flow-through). The effects of treatment on sex ratio, sex organ weights, gonadal histomorphology, breeding behaviour, fertility and on secondary sexual characteristics (nuptial pad size/colour/histomorphology and forelimb width in males) were investigated in sexually mature frogs (6 months post-metamorphosis). In adult females, ovarian histomorphology was analysed in a sub-sample that were sacrificed prior to breeding. In adult males gonadal histomorphology was conducted on a sub-sample of individuals immediately following breeding.

1 Berg, C., Gyllenhammar, I. & Kvarnryd, M. Xenopus tropicalis as a Test System for Developmental and Reproductive Toxicity. *Journal of Toxicology and Environmental Health, Part A* **72**, 219-225, (2009).

2 U.S. EPA. Risks of Linuron Use to Federally Threatened California Red-legged Frog (Rana aurora draytonii). https://www3.epa.gov/pesticides/endanger/litstatus/effects/redleg-frog/linuron/determination.pdf. 2008

3 Grabic, R., Fick, J., Lindberg, R. H., Fedorova, G. & Tysklind, M. Multi-residue method for trace level determination of pharmaceuticals in environmental samples using liquid chromatography coupled to triple quadrupole mass spectrometry. *Talanta* **100**, 183-195, (2012).

4 Jansson, C. & Kreuger, J. Multiresidue Analysis of 95 Pesticides at Low Nanogram/Liter Levels in Surface Waters Using Online Preconcentration and High Performance Liquid Chromatography/Tandem Mass Spectrometry. *Journal of AOAC International* **93**, 1732-1747 (2010).

Figure S1. Measurement of forelimb width and length.

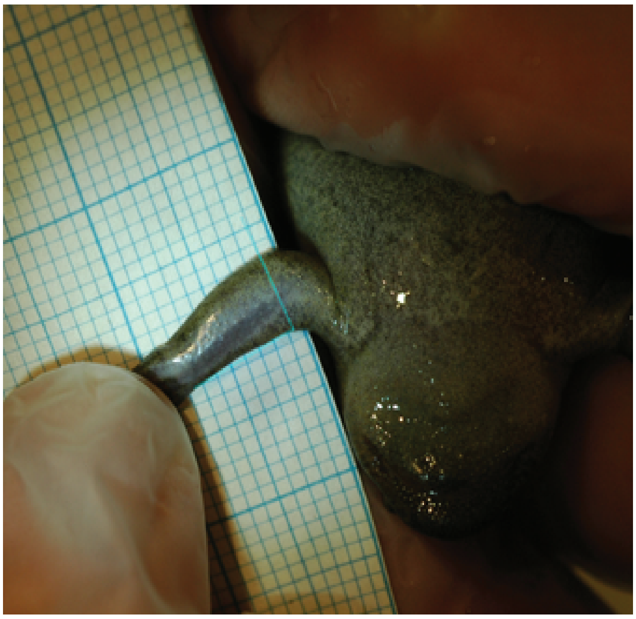

Before the second boosting injection, each male frog was photographed. A piece of graph paper was held beneath the forearm. The camera (Nicon D70, objective AF micro Nikkor 60 mm 1:2:8D) was held by a camera support, approximately 30 cm over a bench, facing it, and a torch was used to illuminate the arm. The forelimb length and width of the male frogs were measured in the photos using an image analysis program, ImageJ. The graph paper beneath the forelimb on the picture was used as a scale. The length was measured on the inside of the forearm, from the bend at the elbow, down to the wrist. The arm width was measured by rotating this drawn line 90°, then moving it to where the “length line” (first measured line described above) ended at the elbow end, and the width was measured at this point.

Figure S2. Measurement of nuptial pad size and colour.

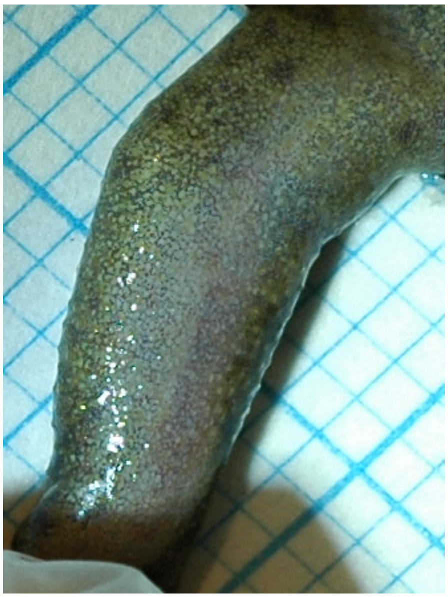

The same photograph was used for forelimb width and nuptial pad measurements. The size and colour intensity of the nuptial pad were analysed using Adobe Photoshop CS6. The nuptial pad was selected using the Quick Selection Tool, and the area (in number of pixels) and colour intensities were recorded. Colour intensity ranged from 0 (black) to 255 (white). Reflections on the arm from water drops have an intense white colour which interferes with the colour intensity measurements. To eliminate this artefact the reflections were removed using the Spot Healing Brush Tool. This tool allows the removal of the reflections by replacing these areas with a composite colour sampled from the skin surrounding the reflection. For each photo the area was calculated by comparing the number of pixels in the selected part with the number of pixels in a 1x2 mm selected area in the graph paper. Each photograph was analysed twice without knowledge of treatment and the mean of these measurements was used for data analysis.

Supplementary methods S3 – Fertility determination

Two factors were taken into account during optimisation:

1. Practicality: Development over 20 hours after amplexus had ceased (~26 hours after initiation of amplexus) allowed time to remove the fertilized eggs and get the tanks ready for the next batch of eggs for the following breeding trials (depending on amplexus behaviour, there could be as little as 30 minutes between the two).
2. Optimal timing: As embryo development starts directly at fertilization, 20 hours after amplexus ceased fertilized oocytesd should have reached at least Nieuwkoop and Faber 25 to 33 (depending on temperature) and fertilized eggs should be obvious/easy to identify.

Fertilisation success was measured at 18, 19, 20 and 21 hours after amplexus ceased. Variability in fertility rate between aquarias was observed, with higher levels in aquaria 1 than aquaria 2. However, in both In aquarium 1 (Figure 1) and aquarium 2 (Figure 2) 20 hours was shown to be sufficient to capture the vast majority of the fertilisation that had occurred.

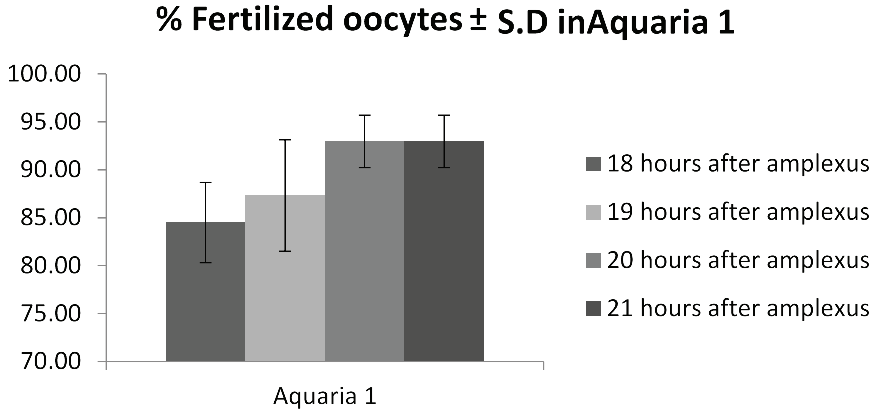

Figure 1. Percentage fertilization success in aquarium 1. Three separate petri dishes were cultivated over night in water bath and analyzed 18, 19, 20 and 21 hours after amplexus ceased. Data presented as mean ± S.D.

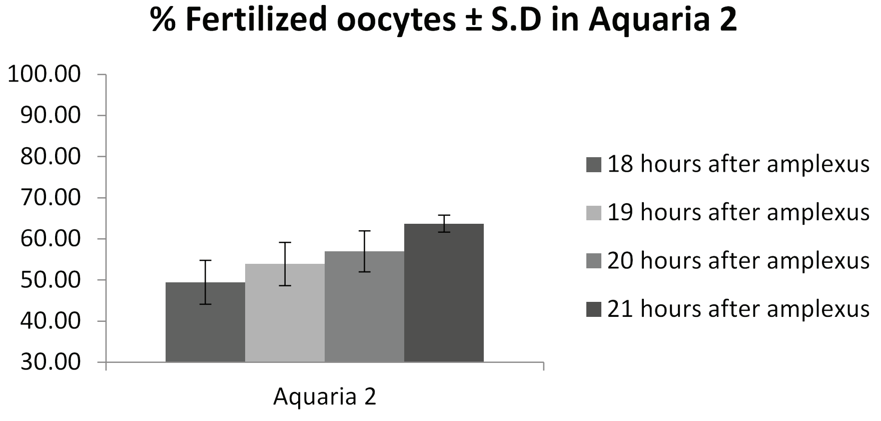

Figure 2. Percentage fertilization success in aquarium 2. Three separate petri dishes was cultivated over night in water bath and analyzed 18, 19, 20 and 21 hours after amplexus ceased. Data presented as mean ± S.D.

Supplementary methods S4 – Adult male histomorphology

For tests histomorphology, digital photos of a secton cut through the centre of the right tests were captured with a photo-micrographic camera (Leica DFC 550, Leica AB, Kista). For each secton analysed, a grid was overlaid and all seminiferous tubules that contained crossing gridlines (spacing: 0.7 mm) were selected for analysis (3-23 tubules, depending on the size of the testis). For each seminiferous tubule analysed, the number of germ cell nests (cyst-like structures within the luminal margins of the seminiferous tubule) and each nest was classified according to the most mature cell type observed: spermatocytes, spermatids or spermatozoa, using established criteria[1]. In addition, the number of spermatogonia per tubule and the number of spermatocytes in the largest spermatocyte nest in each tubule (2-20 nests per tubule) were counted. The amount of spermatozoa in the lumen of the tubule was assessed and assigned a score number (spermatozoa: 1 = tubules with little/no spermatozoa, 2 = tubules with spermatozoa in half of the lumen, 3 = tubules with lumen filled with spermatozoa)[2]. Testis and tubule diameter length and width (average calculated for data analysis) and the total number of seminiferous tubules were recorded. Testcular morphology was compared between treatments using mean values for each measured endpoint across tubules within individuals. Analysis was done directly or using ImageJ software (Natonal Insttute of Health, Bethesda, MD, USA) where appropriate. All slides were analysed without knowledge of exposure group.

1. Kalt, M. R. Morphology and kine ics of spermatogenesis in Xenopus laevis. *Journal of Experimental Zoology* **195**, 393-407, doi:10.1002/jez.1401950306 (1976)
2. Gyllenhammar, I., Holm, L., Eklund, R. & Berg, C. Reproductive toxicity in Xenopus tropicalis after dvelopmental exposure to environmental concentrations of ethynylestradiol. Aquatic Toxicology 91, 171-178 (2009)

Photomicrographs of gonadal sections from adult *Xenopus tropicalis* showing (A) ovarian oocytes in various developmental stages, (B) a seminiferous tubule with the luminal space filled with spermatozoa (SZ) (Score 3), (C) a seminiferous tubule with spermatozoa occupying approximately half of the luminal space (Score 2), and (D) a seminiferous tubule lacking luminal spermatozoa (Score 1). SPG = spermatogonia, SC = spermatocytes, ST = spermatids, L = lumen.

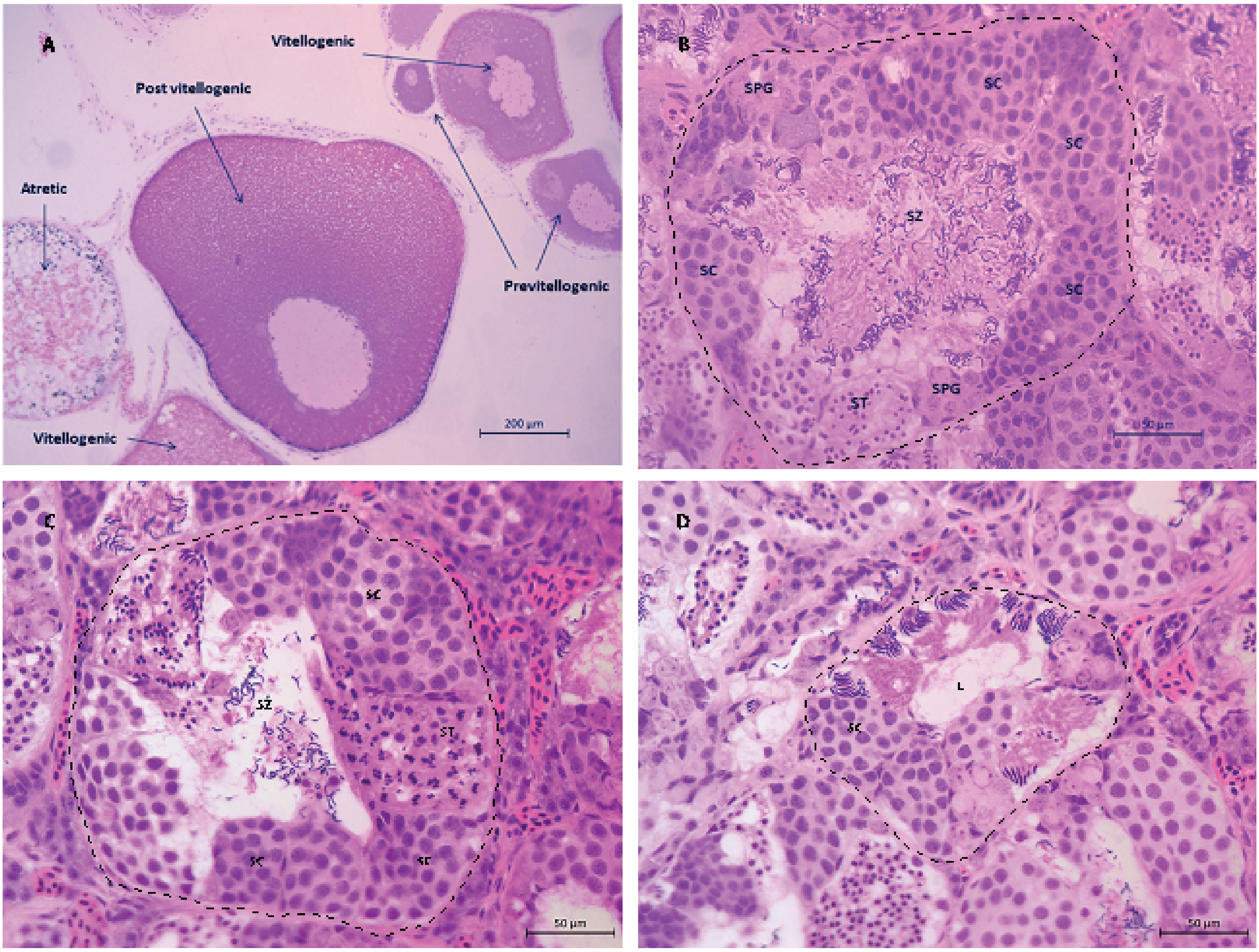

Figure S3. Photomicrographs of arm sections from adult *Silurana tropicalis* showing (A) an arm showing many keratinised hooks (KH) and breeding glands/special mucous glands (BG) at 25 % through the arm (x100), (B) an arm showing a BG and KH at 50 % through the arm (x400), (C) an arm showing normal mucous glands/serous glands (MG) and no KH at 75 % through the arm (x200), and (D), an arm showing a MG at 50 % through the arm (x400).

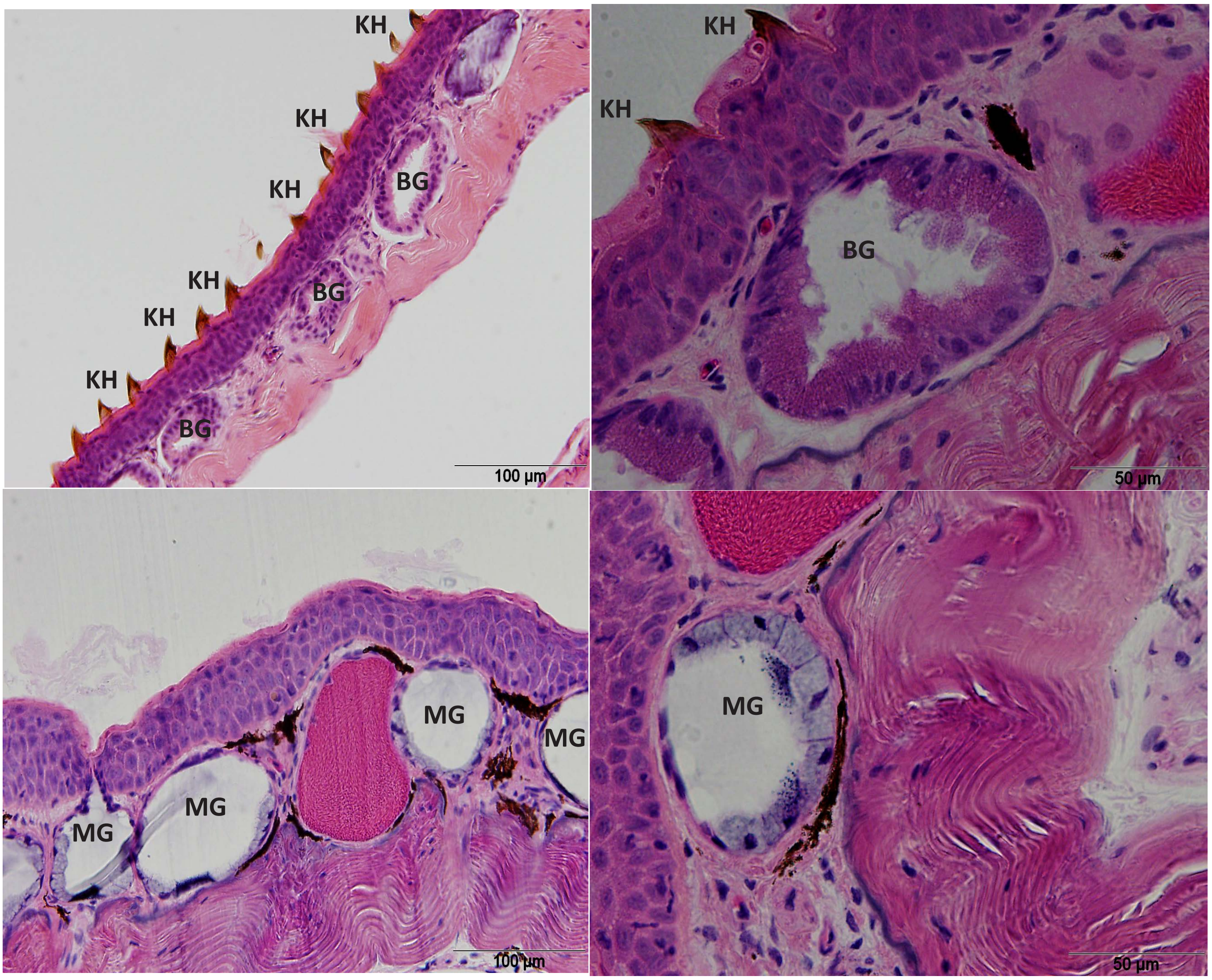

Supplementary methods S5 – Molecular methods

Tissue samples were homogenized using a Bullet Blender® Storm 24 (speed 8, 1 min, 0.5 mm zirconium oxide bullets: Next Advance, Inc. New York USA) in sample lysis solution (30 µl). RNA was extracted from the brains, gonads and arms using TRI reagent (Sigma-Aldrich) according to the manufacturer's instructions. RNA concentration and purity were assessed with a NanoDrop ND-1000 Spectrophotometer (NanoDrop Technologies, Wilmington, USA). cDNA was synthesised according to manufacturer's instructions from 2 µg of total RNA treated with RQ1 DNase (Promega, Southampton, UK) using random hexamers (MWG-Biotech) and M-MLV reverse transcriptase (Promega).

All designed primer pairs were optimised using standard curves and melt curve analysis was used to determine amplicon specificity. The 20 µl qPCR reaction mixture consisted of 50 % iQ™ SYBR®Green Supermix (Bio-Rad Laboratories Inc. CA USA), 0.2 µM of forward and reverse primers and 0.05, 0.1, 0.5 or 1.0 ng/µl cDNA. The qPCR program included an enzyme activation step for 3 minutes at 95 °C and 40-45 cycles of 15 seconds at 95 °C and 30 seconds at primer specific annealing temperature. A melt curve analysis running from 55 °C to 95 °C generated a dissociation curve to confirm the presence of a single amplicon (Rotor-Gene 6000, Qiagen, Hilden, Germany). Primer sequences were available for *ar*[1] and primers for *gr* were designed with the Beacon Designer software (Premier Biosoft International, Palo Alto, CA). All primers were purchased from MWG-Eurofin (Ebersburg, Germany) and verified using BLAST for alignment to the specific target genes. All designed primer pairs were optimised for annealing temperature. The expression of all gene targets were analysed with the CX96 ™ Real-Time System (C1000 ™ thermo cycler, Bio-Rad Laboratories, CA). The 15 µl qPCR reaction mixture consisted of 50% iQ™ SYBR®Green Supermix (Bio-Rad Laboratories Inc. CA USA), 10 µM of forward and reverse primers and 0.001-35 ng cDNA. For *ar*, the qPCR program included an enzyme activation step for 3 minutes at 95°C and 45 cycles of 15 seconds at 95 °C/30 seconds at 60^0C. Details on primer sequences and qPCR assay conditions are shown in Supplementary, Table S1.

1 Koressaar, T. & Remm, M. Enhancements and modifications of primer design program Primer3. *Bioinformatics (Oxford, England)* **23**, 1289-1291 (2007).

Table S1. Gene specific qPCR primers for *S.tropicalis.*

Target gene	Primer (5' – 3') sense	Primer (5' – 3') antisense	Accession no.	Ta (ºC)
ar[a]	ACCTGGTGTTTAATGAGTATCGC	CAGGAATAATGCTAAAGAGAAGGAG	XM_002941842.2	60
gr	CCATCTCACAGCATCAG	ACCTATTCCAGCCTTCT	-	60
rpl8[b]	CCCTCAACCATCAGGAGAGA	TCTTTGTACCACGCAGACGA	BC059744	62

[a]ar primers, Koressar and Remm, *Bioinformatics*

[b]rpls8 primers, Langlois et al. 2010, Gen Comp Endocrin 417-427

Ta = annealing temperature.

Table S2. Covariates included in models and justification for inclusion for modelling

Breeding outcomes (Y variables)	Covariates	Model Inclusion?	Justification
**Breeding behaviour**			
Achieving Amplexus	interval	Yes	Male frogs are competing for the female, therefore frogs could plausibly be affected by the time since their last breeding (NB: there is no interval value for breeding 1)
competing frogs 1 & 2	female weight	No	Male frogs are competing for the same female, it is not likely the weight of that female would affect the competition between the male frogs.
	fecundity	No	Should not be included in model as occurs after a frog has won or lost
	treatment	Yes	The treatment of the competing frog could plausibly affect the outcome of the win-lose (NB: does not apply to breeding 1 as within treatments).
**Breeding outcomes for successful frog**			
Fertility	interval	Y	male frogs fertility could plausibly be affected by the time since their last breeding (there is no interval value for breeding 1)
non-competing frog 1 only	female weight	Y	female weight could have an impact on the quality of the eggs laid and therefore the proportion that could be fertilised
	fecundity	Y	The total number of eggs laid could be related to the quality of those eggs, and therefore could affect the proportion that are able to be fertilised
	Treatment	N	Only the fertility of the winning frog is analysed, this is already split by treatment in the analyses

Table S3. Variable groups for modelling

**Morhpology**			**Nuptial pad morphology**			**Gonadal morphology**			**Nuptial pad histomorphology**			**Gonadal histomorphology**			**Androgen Axis**			**Glucocorticoid Axis**		
Variable	*Breeding Trial*		*Variable*	*Breeding Trial*		*Variable*	*Breeding Trial*		*Variable*	*Breeding Trial*		*Variable*	*Breeding Trial*		*Variable*	*Breeding Trial*		*Variable*	*Breeding Trial*	
	1	2		1	2		1	2		1	2		1	2		1	2		1	2
Male weight	Yes	Yes	Dark colour	Yes	Yes	Testis weight	No	Yes	Size serous	No	Yes	N spermatogonia	No	Yes	Testosterone	No	Yes	Corticosterone	No	Yes
Male length	No	Yes	Whole size	Yes	Yes	N tubules	No	Yes	N serous	No	Yes	N spermatocytes	No	Yes	Testis *ar*	No	Yes	Testis *gr*	No	Yes
Forelimb width	Yes	Yes	ProportioNo dark	Yes	Yes				Size SM gland	No	Yes	N germ cell nest	No	Yes	Brain *ar*	No	Yes	Brain *gr*	No	Yes
Forelimb size	Yes	Yes							N SM gland	No	Yes	% Spermatocyte	No	Yes	Forelimb *ar*	No	Yes	Forelimb *gr*	No	Yes
									N Hooks	No	Yes	% Spermatid	No	Yes						
												% Spermatozoa	No	Yes						

Table S4 Models for achieving amplexus in breeding 1. For each variable, "1" refers to frog 1 of the pair and "2" refers to frog 2 of the pair, highlighted indicates significance whereby effects were seen for both frogs in the pair.

Morphology															
Unexposed				Linuron Low				Linuron High				Flutamide			
Percentiles	5.00%	50.00%	95.00%	Percentiles	5.00%	50.00%	95.00%	Percentiles	5.00%	50.00%	95.00%	Percentiles	5.00%	50.00%	95.00%
(Intercept)	-0.50	0.00	0.51	(Intercept)	-0.58	-0.01	0.55	(Intercept)	-0.59	-0.01	0.56	(Intercept)	-1.02	-0.02	1.01
weight 1	-0.21	0.40	1.05	weight 1	-0.92	-0.14	0.61	weight 1	-0.53	0.18	0.89	weight 1	-3.35	-1.27	0.73
weight 2	-1.02	-0.39	0.20	weight 2	-0.63	0.15	0.95	weight 2	-0.91	-0.21	0.50	weight 2	-0.72	1.28	3.32
FLW 1	-1.10	-0.36	0.33	FLW 1	-0.32	0.36	1.13	FLW 1	-0.37	0.45	1.37	FLW 1	-0.42	0.87	2.27
FLW 2	-0.36	0.36	1.09	FLW 2	-1.18	-0.36	0.33	FLW 2	-1.36	-0.43	0.42	FLW 2	-2.17	-0.85	0.39
FL size 1	-0.75	-0.03	0.65	FL size 1	-0.52	0.18	0.93	FL size 1	-1.34	-0.56	0.16	FL size 1	-0.18	1.42	3.22
FL size 2	-0.66	0.04	0.74	FL size 2	-0.95	-0.19	0.54	FL size 2	-0.14	0.57	1.32	FL size 2	-3.16	-1.39	0.23
Nuptial Pad Morphology															
Unexposed				Linuron Low				Linuron High				Flutamide			
Percentiles	5.00%	50.00%	95.00%	Percentiles	5.00%	50.00%	95.00%	Percentiles	5.00%	50.00%	95.00%	Percentiles	5.00%	50.00%	95.00%
(Intercept)	-0.54	-0.01	0.54	(Intercept)	-0.57	0.01	0.58	(Intercept)	-0.62	0.01	0.62	(Intercept)	-1.02	0.00	1.00
Proportion 1	-0.73	-0.08	0.56	Proportion 1	-0.81	-0.12	0.57	Proportion 1	-0.81	-0.14	0.56	Proportion 1	-0.73	0.42	1.59
Proportion 2	-0.57	0.09	0.73	Proportion 2	-0.59	0.11	0.84	Proportion 2	-0.53	0.12	0.82	Proportion 2	-1.59	-0.43	0.70
Colour 1	-0.49	0.27	1.03	Colour 1	-0.11	0.56	1.28	Colour 1	0.52	1.33	2.33	Colour 1	-2.07	-0.31	1.51
Colour 2	-1.04	-0.27	0.48	Colour 2	-1.32	-0.57	0.14	Colour 2	-2.28	-1.35	-0.53	Colour 2	-1.52	0.29	2.03
Size 1	0.02	0.60	1.23	Size 1	-0.52	0.14	0.78	Size 1	0.00	0.67	1.42	Size 1	-0.48	0.92	2.46
Size 2	-1.27	-0.60	-0.01	Size 2	-0.78	-0.15	0.51	Size 2	-1.39	-0.67	-0.02	Size 2	-2.46	-0.91	0.40

Table S5 Models for achieving amplexus in breeding 2 for morphological endpoints. For each variable, "1" refers to frog 1 of the pair and "2" refers to frog 2 of the pair, highlighted indicates significance whereby effects were seen for both frogs in the pair.

Morphology															
Unexposed				Linuron Low				Linuron High				Flutamide			
Percentiles	5.00%	50.00%	95.00%	Percentiles	5.00%	50.00%	95.00%	Percentiles	5.00%	50.00%	95.00%	Percentiles	5.00%	50.00%	95.00%
(Intercept)	-2.47	-0.58	1.21	(Intercept)	-2.58	0.06	2.86	(Intercept)	-2.38	-0.56	1.28	(Intercept)	-0.28	1.82	4.25
Interval1	-1.92	-0.78	0.32	Interval1	-1.04	0.61	2.24	Interval1	1.33	3.07	5.10	Interval1	-2.35	-0.32	1.68
interval2	-1.91	-0.75	0.30	interval2	-1.91	-0.19	1.67	interval2	-0.21	1.04	2.39	interval2	-3.32	-0.69	2.00
weight 1	-2.29	-0.54	1.11	weight 1	-1.93	0.52	3.21	weight 1	1.58	3.60	5.95	weight 1	-2.19	-0.22	1.95
weight 2	-1.81	-0.10	1.62	weight 2	-3.23	-0.82	1.39	weight 2	-2.18	-0.39	1.30	weight 2	-4.68	-2.36	-0.26
SVL1	-2.52	-0.53	1.44	SVL1	0.37	2.10	4.15	SVL1	-3.11	-1.15	0.74	SVL1	-6.10	-2.92	-0.07
SVL2	-2.78	-1.01	0.71	SVL2	-5.22	-2.87	-0.89	SVL2	-3.09	-1.29	0.44	SVL2	0.41	3.30	6.32
FLW 1	-0.12	1.11	2.43	FLW 1	0.39	1.84	3.46	FLW 1	-3.98	-2.32	-0.91	FLW 1	-5.84	-3.01	-0.64
FLW 2	-0.68	0.60	1.85	FLW 2	-1.56	-0.11	1.36	FLW 2	-0.11	1.12	2.63	FLW 2	-2.69	-0.69	1.26
FL size 1	1.05	2.54	4.23	FL size 1	-1.83	-0.28	1.14	FL size 1	-0.66	0.80	2.38	FL size 1	-2.74	-0.05	2.65
FL size 2	-1.16	-0.02	1.07	FL size 2	-4.43	-2.39	-0.61	FL size 2	-1.19	0.12	1.43	FL size 2	-1.82	0.48	2.82
Treatment	-2.87	-0.25	2.26	Treatment	-2.73	0.51	3.70	Treatment	-0.47	1.83	4.18	Treatment	-4.65	-1.02	2.50
Nuptial Pad Morphology															
Unexposed				Linuron Low				Linuron High				Flutamide			
Percentiles	5.00%	50.00%	95.00%	Percentiles	5.00%	50.00%	95.00%	Percentiles	5.00%	50.00%	95.00%	Percentiles	5.00%	50.00%	95.00%
(Intercept)	-2.73	-0.99	0.60	(Intercept)	-2.13	0.68	3.51	(Intercept)	-1.53	-0.22	1.08	(Intercept)	-0.06	1.80	3.85
Interval1	-1.79	-0.80	0.13	Interval1	-3.22	-0.98	1.10	Interval1	0.54	1.63	3.04	Interval1	-2.73	-0.85	1.00
interval2	-1.50	-0.39	0.64	interval2	0.51	2.42	4.65	interval2	-0.36	0.50	1.39	interval2	-2.83	-0.84	1.16
Proportion 1	-1.57	-0.54	0.39	Proportion 1	0.58	2.28	4.35	Proportion 1	-1.13	-0.20	0.69	Proportion 1	-3.12	-0.97	0.91
Proportion 2	-0.04	1.08	2.29	Proportion 2	-3.10	-1.36	0.15	Proportion 2	-0.98	-0.04	0.88	Proportion 2	-2.03	0.41	2.76
Colour 1	-4.53	-2.85	-1.35	Colour 1	-6.70	-4.01	-2.00	Colour 1	-2.16	-1.06	-0.08	Colour 1	-5.49	-2.97	-1.10
Colour 2	1.15	2.73	4.49	Colour 2	0.50	2.32	4.61	Colour 2	0.16	1.06	2.12	Colour 2	0.17	2.61	5.25
Size 1	-0.46	0.65	1.80	Size 1	-2.50	-0.16	2.13	Size 1	-1.17	-0.20	0.72	Size 1	-3.79	-1.77	-0.04
Size 2	-2.29	-1.05	0.12	Size 2	-1.51	0.70	2.69	Size 2	-0.82	0.04	0.99	Size 2	0.25	1.89	4.02
Treatment	-2.94	-0.73	1.48	Treatment	-4.23	-0.70	2.85	Treatment	-0.77	0.94	2.70	Treatment	-5.30	-1.74	1.74

Table S6 Models for achieving amplexus in breeding 2 for nuptial pad histomorphology endpoints. For each variable, "1" refers to frog 1 of the pair and "2" refers to frog 2 of the pair, highlighted indicates significance whereby effects were seen for both frogs in the pair. No. = number, SM = Special Mucous Glands

Nuptial Pad histomorphology															
Unexposed				Linuron Low				Linuron High				Flutamide			
Percentiles	5.00%	50.00%	95.00%	Percentiles	5.00%	50.00%	95.00%	Percentiles	5.00%	50.00%	95.00%	Percentiles	5.00%	50.00%	95.00%
(Intercept)	-3.07	-0.92	1.19	(Intercept)	-1.49	0.63	2.73	(Intercept)	-3.01	-0.21	2.66	(Intercept)	-1.80	0.40	2.71
Interval1	-0.48	0.74	2.03	Interval1	-2.28	-0.71	0.75	Interval1	-2.02	0.12	2.22	Interval1	-3.96	-1.68	0.44
interval2	-1.81	-0.53	0.70	interval2	-0.76	0.74	2.22	interval2	1.56	3.76	6.59	interval2	-3.33	-1.14	0.84
Size serous1	-0.42	0.76	2.14	Size serous1	-1.93	-0.45	0.78	Size serous1	-2.25	-0.51	1.20	Size serous1	-2.94	-0.06	2.81
Size serous2	-2.93	-1.01	0.58	Size serous2	-0.37	0.90	2.41	Size serous2	-2.32	-0.18	1.98	Size serous2	-3.98	-1.64	0.44
No. serous1	-1.22	0.13	1.60	No. serous1	-1.25	0.16	1.63	No. serous1	-1.63	0.42	2.59	No. serous1	-5.48	-2.53	-0.13
No. serous2	-3.61	-1.55	0.40	No. serous2	-1.79	-0.34	1.03	No. serous2	-1.58	0.37	2.38	No. serous2	-4.42	-1.90	0.53
No. SM1	-1.68	-0.11	1.56	No. SM1	-2.97	-1.30	0.20	No. SM1	-3.26	-0.50	2.32	No. SM1	-3.28	-0.38	2.53
No. SM2	-0.88	1.07	3.07	No. SM2	-0.39	1.23	3.02	No. SM2	-2.04	0.71	3.38	No. SM2	-3.55	-1.04	1.53
No. hooks1	0.37	2.00	3.71	No. hooks1	-1.67	0.11	1.91	No. hooks1	-5.03	-2.43	-0.08	No. hooks1	-6.74	-3.97	-1.28
No. hooks2	-5.03	-2.80	-0.73	No. hooks2	-2.37	-0.65	1.05	No. hooks2	-0.50	1.99	4.72	No. hooks2	-1.81	1.07	3.91
Treatment	-3.84	-1.06	1.71	Treatment	-3.84	-0.76	2.30	Treatment	-3.47	-0.20	2.96	Treatment	-3.65	0.07	3.57

Table S7 Models for achieving amplexus in breeding 2 for testicular morphology endpoints. For each variable, "1" refers to frog 1 of the pair and "2" refers to frog 2 of the pair. Red = positive association for both individuals in the pair and thus excluded from results reporting.

Testis morphology															
Unexposed				Linuron Low				Linuron High				Flutamide			
Percentiles	5.00%	50.00%	95.00%	Percentiles	5.00%	50.00%	95.00%	Percentiles	5.00%	50.00%	95.00%	Percentiles	5.00%	50.00%	95.00%
(Intercept)	-4.38	-1.81	0.55	(Intercept)	-4.87	-0.85	2.64	(Intercept)	-3.74	-1.27	1.05	(Intercept)	-4.20	-0.70	2.73
Interval1	-3.69	-1.48	0.57	Interval1	-1.18	0.36	1.86	Interval1	0.66	2.87	5.62	Interval1	-2.77	-0.39	1.89
interval2	-1.93	-0.44	1.08	interval2	-1.27	0.79	2.93	interval2	-2.20	-0.34	1.24	interval2	-0.94	2.28	6.07
testis_wgt1	-10.64	-3.53	2.91	testis_wgt1	0.13	2.31	4.94	testis_wgt1	-3.63	-1.39	0.51	testis_wgt1	0.82	5.55	10.95
testis_wgt2	-4.47	-1.71	0.49	testis_wgt2	-19.33	-5.94	4.84	testis_wgt2	-1.49	0.21	2.01	testis_wgt2	0.23	2.36	4.96
No_tubules1	-0.58	0.84	2.37	No_tubules1	0.09	2.23	4.53	No_tubules1	-1.24	0.18	1.42	No_tubules1	-4.95	-2.39	-0.05
No_tubules2	-2.51	-0.63	1.09	No_tubules2	-3.67	-1.58	-0.32	No_tubules2	-1.85	-0.25	1.15	No_tubules2	-2.04	-0.04	2.12
Treatment	-3.30	-0.28	2.75	Treatment	-3.14	0.28	3.58	Treatment	-0.52	2.28	5.33	Treatment	-4.42	-0.59	3.08

Table S8 Models for achieving amplexus in breeding 2 for testicular histomorphology endpoints. For each variable, "1" refers to frog 1 of the pair and "2" refers to frog 2 of the pair.

Testicular histomorphology															
Unexposed				Linuron Low				Linuron High				Flutamide			
Percentiles	5.00%	50.00%	95.00%	Percentiles	5.00%	50.00%	95.00%	Percentiles	5.00%	50.00%	95.00%	Percentiles	5.00%	50.00%	95.00%
(Intercept)	-5.87	-2.74	0.11	(Intercept)	-0.77	2.60	6.38	(Intercept)	-4.26	-1.12	1.81	(Intercept)	-0.31	2.99	6.61
Interval1	-5.26	-2.45	0.17	Interval1	-2.23	-0.04	2.18	Interval1	-0.69	2.44	5.78	Interval1	-4.22	-1.29	1.72
interval2	-0.88	1.30	3.77	interval2	-0.63	1.72	4.19	interval2	-3.94	-1.27	1.13	interval2	-1.07	2.46	6.06
No. spermatogonia1	-3.81	-0.65	2.39	No. spermatogo	0.27	2.56	5.45	No. spermatogo	-1.69	1.37	4.48	No. spermatogo	0.77	3.48	6.60
No. spermatogonia2	-5.95	-3.11	-0.54	No. spermatogo	-5.92	-2.92	-0.13	No. spermatogo	-5.72	-2.63	0.57	No. spermatogo	-1.61	1.94	5.57
No germnest1	-2.60	0.31	3.34	No germnest1	-1.94	1.44	4.64	No germnest1	-4.46	-1.39	1.69	No germnest1	-3.64	-0.04	3.63
No germnest2	-1.04	1.83	4.71	No germnest2	-5.11	-2.09	0.94	No germnest2	-1.91	1.07	3.91	No germnest2	-4.43	-1.03	2.38
% spermatocyte1	-3.62	-0.19	3.10	% spermatocyte	-2.27	0.69	3.79	% spermatocyte	-3.26	0.53	4.16	% spermatocyte	-2.87	0.51	4.03
% spermatocyte2	-3.88	-0.62	2.71	% spermatocyte	-3.92	-0.97	1.94	% spermatocyte	-3.71	0.18	3.95	% spermatocyte	-3.62	-0.21	3.25
% spermatid1	-3.25	-0.19	2.87	% spermatid1	-1.04	2.04	5.16	% spermatid1	-2.11	1.08	4.42	% spermatid1	-2.41	0.42	3.18
% spermatid2	-4.93	-1.70	1.29	% spermatid2	-2.76	0.26	3.39	% spermatid2	-2.51	0.45	3.31	% spermatid2	-3.74	-0.60	2.44
% spermatozoa1	-2.90	0.35	3.84	% spermatozoa1	-5.66	-2.58	0.39	% spermatozoa1	-4.31	-1.66	0.77	% spermatozoa1	-4.09	-0.85	2.49
% spermatozoa2	0.32	3.38	6.52	% spermatozoa2	-2.14	0.74	3.65	% spermatozoa2	-3.23	-0.46	2.07	% spermatozoa2	-3.07	1.22	5.40
No. spermatocytes1	-1.26	1.44	4.21	No. spermatocy	-3.80	-0.47	2.83	No. spermatocy	-3.36	-0.38	2.46	No. spermatocy	-5.54	-2.11	1.29
No. spermatocytes2	-4.23	-0.95	2.42	No. spermatocy	-2.36	0.61	3.58	No. spermatocy	-2.45	0.62	3.72	No. spermatocy	-2.37	0.86	4.16
Treatment	-4.10	-0.52	3.05	Treatment	-4.38	-0.67	3.01	Treatment	-1.85	1.33	4.74	Treatment	-4.40	-0.56	3.29

Table S9 Models for achieving amplexus in breeding 2 for androgens/androgen receptor endpoints. For each variable, "1" refers to frog 1 of the pair and "2" refers to frog 2 of the pair.

Androgens and androgen receptor mRNA															
Unexposed				Linuron Low				Linuron High				Flutamide			
Percentiles	5.00%	50.00%	95.00%	Percentiles	5.00%	50.00%	95.00%	Percentiles	5.00%	50.00%	95.00%	Percentiles	5.00%	50.00%	95.00%
(Intercept)	-2.56	-0.76	1.00	(Intercept)	-1.51	0.71	2.91	(Intercept)	0.05	2.78	5.60	(Intercept)	-0.23	2.15	4.70
Interval1	-1.47	-0.44	0.48	Interval1	-2.33	-0.74	0.66	Interval1	0.84	2.74	5.30	Interval1	-2.89	-0.54	1.70
interval2	-0.93	0.07	1.10	interval2	-0.37	0.93	2.56	interval2	-2.08	-0.43	1.03	interval2	-1.23	0.87	2.99
tesosterone1	-0.29	0.77	1.84	tesosterone1	-0.27	0.73	1.79	tesosterone1	-0.95	0.58	2.12	tesosterone1	-3.65	-1.16	1.16
tesosterone2	-2.06	-0.95	0.15	tesosterone2	-1.60	-0.55	0.42	tesosterone2	-2.39	-1.06	0.23	tesosterone2	-2.50	0.00	2.69
testis_AR1	-0.84	0.08	1.02	testis_AR1	-1.36	-0.34	0.61	testis_AR1	-4.57	-2.32	-0.50	testis_AR1	-1.00	1.32	3.94
testis_AR2	-1.14	-0.16	0.78	testis_AR2	-1.09	-0.08	0.83	testis_AR2	0.63	2.15	3.98	testis_AR2	-2.95	-0.69	1.60
brain_AR1	-1.55	-0.47	0.48	brain_AR1	-3.62	-1.92	-0.67	brain_AR1	-2.17	-0.48	1.13	brain_AR1	-6.26	-3.54	-1.11
brain_AR2	0.01	0.94	1.97	brain_AR2	0.29	1.25	2.36	brain_AR2	-1.85	-0.38	1.04	brain_AR2	-0.04	2.22	5.36
forelimb AR1	-1.87	0.77	3.44	forelimb AR1	-1.55	-0.01	1.44	forelimb AR1	-0.47	1.09	2.73	forelimb AR1	-3.31	-0.69	1.48
forelimb AR2	-5.61	-2.85	-0.37	forelimb AR2	-1.02	0.47	2.11	forelimb AR2	-2.33	-0.92	0.38	forelimb AR2	-1.18	1.44	4.51
Treatment	-2.18	0.29	2.70	Treatment	-3.81	-1.06	1.64	Treatment	-5.10	-2.03	1.19	Treatment	-5.11	-1.39	2.22

Table S10 Models for achieving amplexus in breeding 2 for corticosterone/glucocorticoid receptor endpoints. For each variable, "1" refers to frog 1 of the pair and "2" refers to frog 2 of the pair.

Corticosterone and glucocorticoid receptor															
Unexposed				Linuron Low				Linuron High				Flutamide			
Percentiles	5.00%	50.00%	95.00%	Percentiles	5.00%	50.00%	95.00%	Percentiles	5.00%	50.00%	95.00%	Percentiles	5.00%	50.00%	95.00%
(Intercept)	-2.62	-0.51	1.59	(Intercept)	-1.94	0.31	2.52	(Intercept)	-1.34	1.43	4.34	(Intercept)	-1.57	1.18	4.13
Interval1	-1.70	-0.21	1.34	Interval1	-3.15	-1.01	1.09	Interval1	1.03	3.03	5.25	Interval1	-3.58	-0.28	3.13
interval2	-3.53	-1.53	0.19	interval2	-1.20	1.02	3.25	interval2	-2.23	-0.09	2.04	interval2	-3.66	-1.03	1.52
forelimb GR1	-0.72	0.95	2.73	forelimb GR1	-1.57	-0.58	0.32	forelimb GR1	-2.12	-0.51	1.04	forelimb GR1	-4.94	-2.13	0.66
forelimb GR2	1.41	3.02	4.94	forelimb GR2	-0.49	0.52	1.58	forelimb GR2	-2.11	-0.63	0.77	forelimb GR2	-5.43	-2.29	0.55
brain_GR1	-2.21	-0.89	0.27	brain_GR1	-2.52	-1.08	0.23	brain_GR1	-0.36	1.61	3.64	brain_GR1	-3.01	-0.12	2.75
brain_GR2	-3.47	-1.40	0.32	brain_GR2	0.20	1.45	2.94	brain_GR2	-4.06	-2.18	-0.36	brain_GR2	1.39	4.12	7.43
testis_GR1	-1.74	-0.37	1.01	testis_GR1	0.46	1.69	3.25	testis_GR1	-2.26	-0.30	1.62	testis_GR1	-3.03	-0.38	2.29
testis_GR2	-3.03	-1.49	-0.32	testis_GR2	-2.32	-0.89	0.26	testis_GR2	-0.48	1.19	3.04	testis_GR2	-0.89	2.12	5.28
corticosterone1	-5.65	-3.60	-1.93	corticosterone1	-1.09	-0.09	0.96	corticosterone1	-4.60	-2.06	-0.39	corticosterone1	0.26	2.52	5.20
corticosterone2	0.90	0.41	1.79	corticosterone2	-0.55	0.43	1.42	corticosterone2	0.29	2.58	5.29	corticosterone2	-4.10	-1.40	1.01
Treatment	-1.43	1.62	4.50	Treatment	-3.25	-0.50	2.17	Treatment	-3.68	-0.48	2.72	Treatment	-5.05	-1.32	2.31

Table S11 Models for fertility in breeding 1. No. = number.

Morphology															
Unexposed				Linuron Low				Linuron High				Flutamide			
Percentiles	5.00%	50.00%	95.00%	Percentiles	5.00%	50.00%	95.00%	Percentiles	5.00%	50.00%	95.00%	Percentiles	5.00%	50.00%	95.00%
(Intercept)	-0.42	-0.09	0.25	(Intercept)	-1.05	-0.62	-0.16	(Intercept)	-0.73	-0.38	-0.03	(Intercept)	-1.19	-0.54	0.12
Egg_no	-0.10	0.27	0.65	Egg_no	0.04	0.57	1.08	Egg_no	0.14	0.51	0.89	Egg_no	-0.38	0.62	1.58
weight	-0.34	0.05	0.44	weight	-1.19	-0.52	0.15	weight	-0.86	-0.44	-0.03	weight	-1.26	-0.31	0.76
FLW	-0.22	0.23	0.69	FLW	0.12	0.61	1.08	FLW	-0.54	-0.02	0.46	FLW	-0.66	0.34	1.25
FL size	-0.41	0.06	0.54	FL size	0.01	0.60	1.18	FL size	-0.11	0.37	0.86	FL size	-1.19	0.30	1.58
Nuptial Pad morphology															
Unexposed				Linuron Low				Linuron High				Flutamide			
Percentiles	5.00%	50.00%	95.00%	Percentiles	5.00%	50.00%	95.00%	Percentiles	5.00%	50.00%	95.00%	Percentiles	5.00%	50.00%	95.00%
(Intercept)	-0.41	-0.08	0.25	(Intercept)	-1.06	-0.64	-0.22	(Intercept)	-0.75	-0.38	0.00	(Intercept)	-1.11	-0.53	0.07
Egg_no	-0.10	0.27	0.65	Egg_no	0.01	0.44	0.90	Egg_no	0.16	0.57	0.97	Egg_no	-0.52	0.27	1.08
Proportion	-0.30	0.04	0.40	Proportion	0.38	0.87	1.39	Proportion	-0.19	0.22	0.62	Proportion	-0.50	0.41	1.28
Colour	-0.29	0.08	0.45	Colour	-0.32	0.15	0.64	Colour	-0.06	0.30	0.68	Colour	-0.39	0.65	1.68
Size	-0.25	0.14	0.54	Size	-0.59	-0.15	0.30	Size	-0.26	0.13	0.54	Size	-0.27	0.58	1.37

Table S12 Models for fertility in breeding 2 for morphological endpoints. No. = number

Morphology															
Unexposed				Linuron Low				Linuron High				Flutamide			
Percentiles	5.00%	50.00%	95.00%	Percentiles	5.00%	50.00%	95.00%	Percentiles	5.00%	50.00%	95.00%	Percentiles	5.00%	50.00%	95.00%
(Intercept)	-0.99	-0.31	0.37	(Intercept)	-1.07	-0.62	-0.15	(Intercept)	-0.33	0.04	0.41	(Intercept)	-0.78	0.08	0.96
Egg_no	-1.11	-0.23	0.66	Egg_no	0.04	0.70	1.49	Egg_no	-0.13	0.33	0.81	Egg_no	-1.57	0.50	2.55
Interval1	-1.07	-0.12	0.82	Interval1	-0.06	0.46	1.04	Interval1	-0.55	-0.12	0.30	Interval1	-1.24	-0.12	0.94
weight	-1.72	0.12	2.01	weight	-0.81	-0.15	0.48	weight	-0.93	-0.11	0.66	weight	-1.91	-0.65	0.61
SVL	-1.89	0.18	2.21	SVL	-0.97	-0.39	0.15	SVL	-0.73	-0.05	0.62	SVL	-1.12	0.16	1.48
FLW	-1.04	-0.08	0.85	FLW	-0.89	-0.25	0.44	FLW	-0.77	-0.29	0.20	FLW	-1.06	0.50	2.06
FL size	-1.35	-0.41	0.52	FL size	-0.88	-0.32	0.18	FL size	-0.37	0.21	0.85	FL size	-1.86	0.28	2.39
Nuptial Pad Morphology															
Unexposed				Linuron Low				Linuron High				Flutamide			
Percentiles	5.00%	50.00%	95.00%	Percentiles	5.00%	50.00%	95.00%	Percentiles	5.00%	50.00%	95.00%	Percentiles	5.00%	50.00%	95.00%
(Intercept)	-0.92	-0.37	0.18	(Intercept)	-1.05	-0.59	-0.10	(Intercept)	-0.29	0.05	0.38	(Intercept)	-1.25	-0.22	0.87
Egg_no	-0.89	-0.01	0.93	Egg_no	0.10	0.64	1.20	Egg_no	-0.09	0.31	0.73	Egg_no	-0.49	0.53	1.61
Interval1	-1.02	-0.25	0.44	Interval1	-0.04	0.51	1.03	Interval1	-0.36	0.17	0.66	Interval1	-0.65	0.20	1.04
Proportion	-0.98	-0.25	0.48	Proportion	-0.36	0.28	0.91	Proportion	0.17	0.63	1.12	Proportion	-1.38	0.77	2.72
Colour	-0.81	0.03	0.84	Colour	-0.24	0.43	1.10	Colour	-0.69	-0.24	0.19	Colour	-1.71	1.36	4.05
Size	-0.36	0.25	0.91	Size	-1.21	-0.46	0.30	Size	-0.99	-0.44	0.14	Size	-1.04	-0.02	1.05

Table S13 Models for fertility in breeding 2 for nuptial pad/testicular histomorphology and testicular morphology. No. = number

Nuptial pad histomorphology															
Unexposed				Linuron Low				Linuron High				Flutamide			
Percentiles	5.00%	50.00%	95.00%	Percentiles	5.00%	50.00%	95.00%	Percentiles	5.00%	50.00%	95.00%	Percentiles	5.00%	50.00%	95.00%
(Intercept)	-0.97	-0.31	0.33	(Intercept)	-1.00	-0.43	0.15	(Intercept)	-0.40	0.06	0.48	(Intercept)	-0.67	0.31	1.26
Egg_no	-1.22	-0.34	0.55	Egg_no	-0.11	0.73	1.61	Egg_no	-0.85	-0.07	0.62	Egg_no	-0.60	0.63	1.79
Interval1	-1.26	-0.42	0.54	Interval1	-0.59	0.26	1.11	Interval1	-0.89	-0.24	0.46	Interval1	-0.70	0.28	1.22
Size serous	-0.89	-0.09	0.63	Size serous	-0.79	0.11	0.97	Size serous	-0.23	0.47	1.18	Size serous	-0.75	0.24	1.25
No. serous	-0.58	0.13	0.84	No. serous	-0.55	0.66	1.81	No. serous	-0.75	-0.21	0.29	No. serous	-1.82	0.51	2.70
No. SM	-0.85	0.45	1.70	No. SM	-1.14	0.11	1.26	No. SM	-0.87	0.50	1.77	No. SM	-1.81	0.24	2.10
No. hooks	-1.97	-0.57	0.97	No. hooks	-1.04	-0.12	0.84	No. hooks	-1.44	-0.45	0.55	No. hooks	-1.67	-0.17	1.44
Testicular Morphology															
Unexposed				Linuron Low				Linuron High				Flutamide			
Percentiles	5.00%	50.00%	95.00%	Percentiles	5.00%	50.00%	95.00%	Percentiles	5.00%	50.00%	95.00%	Percentiles	5.00%	50.00%	95.00%
(Intercept)	-2.57	0.06	2.55	(Intercept)	-1.09	-0.31	0.54	(Intercept)	-0.41	0.43	1.29	(Intercept)	-2.72	-0.02	2.53
Egg_no	-2.56	-0.80	0.92	Egg_no	-0.14	0.72	1.55	Egg_no	-1.49	-0.35	0.74	Egg_no	-1.81	0.52	2.92
Interval1	-3.30	-0.67	1.88	Interval1	-0.21	0.47	1.11	Interval1	-1.54	-0.52	0.53	Interval1	-0.96	0.27	1.49
testis weight	-6.74	1.63	9.21	testis weight	-1.01	0.07	1.18	testis weight	-1.08	0.24	1.56	testis weight	-4.31	0.98	6.24
No. tubules	-1.26	-0.16	0.93	No. tubules	-0.74	0.05	0.85	No. tubules	-0.80	0.12	0.99	No. tubules	-1.66	0.11	1.81
Testicular histomorphology															
Unexposed				Linuron Low				Linuron High				Flutamide			
Percentiles	5.00%	50.00%	95.00%	Percentiles	5.00%	50.00%	95.00%	Percentiles	5.00%	50.00%	95.00%	Percentiles	5.00%	50.00%	95.00%
(Intercept)	-1.44	-0.34	0.71	(Intercept)	-0.89	-0.24	0.42	(Intercept)	-0.52	0.40	1.31	(Intercept)	-1.17	0.25	1.70
Egg_no	-2.36	-0.72	0.80	Egg_no	-0.04	0.68	1.38	Egg_no	-1.45	-0.28	0.89	Egg_no	-2.14	0.52	3.25
Interval1	-3.27	-1.12	1.15	Interval1	-0.47	0.25	0.97	Interval1	-1.66	-0.51	0.70	Interval1	-2.22	-0.01	2.26
No spermatogonia	-1.91	0.19	2.28	No spermatogor	-1.52	-0.41	0.67	No spermatogor	-0.96	0.08	1.11	No spermatogor	-1.87	0.35	2.50
No spermatocytes	-2.58	-0.27	2.04	No spermatocyt	-0.58	0.55	1.69	No spermatocyt	-1.06	0.09	1.25	No spermatocyt	-2.17	0.01	2.28
NB: testis histopathology variables split into two models due to low n number for these endpoints															
Unexposed				Linuron Low				Linuron High				Flutamide			
Percentiles	5.00%	50.00%	95.00%	Percentiles	5.00%	50.00%	95.00%	Percentiles	5.00%	50.00%	95.00%	Percentiles	5.00%	50.00%	95.00%
(Intercept)	-1.72	-0.39	1.01	(Intercept)	-0.82	-0.19	0.44	(Intercept)	-0.46	0.42	1.28	(Intercept)	-0.70	0.48	1.58
Egg_no	-4.06	-0.68	2.94	Egg_no	0.18	0.85	1.54	Egg_no	-1.44	-0.32	0.85	Egg_no	-2.04	0.52	3.04
Interval1	-3.06	-0.92	1.25	Interval1	-0.33	0.31	0.98	Interval1	-1.67	-0.53	0.57	Interval1	-1.76	0.25	2.30
% spermatid	-1.90	0.05	1.83	spermatid1	-0.42	0.28	0.99	spermatid1	-1.07	-0.09	0.94	spermatid1	-1.64	-0.11	1.44
% spermatozoa	-1.76	0.09	1.99	spermatozoa1	-1.39	-0.57	0.25	spermatozoa1	-1.05	-0.10	0.97	spermatozoa1	-2.07	0.29	2.62
NB: testis histopathology variables split into two models due to low n number for these endpoints															

Table S14 Models for fertility in breeding 2 for androgens/corticosterone and androgen/glucocorticoid receptor.

Androgens and androgen receptor															
Unexposed				Linuron Low				Linuron High				Flutamide			
Percentiles	5.00%	50.00%	95.00%	Percentiles	5.00%	50.00%	95.00%	Percentiles	5.00%	50.00%	95.00%	Percentiles	5.00%	50.00%	95.00%
(Intercept)	-1.02	-0.48	0.11	(Intercept)	-0.93	-0.48	-0.04	(Intercept)	-0.26	0.10	0.48	(Intercept)	-0.81	0.18	1.13
Egg_no	-0.60	0.12	0.83	Egg_no	0.16	0.63	1.15	Egg_no	-0.31	0.07	0.45	Egg_no	-0.27	0.82	1.95
Interval1	-0.72	-0.11	0.50	Interval1	-0.40	0.16	0.76	Interval1	-0.73	-0.27	0.17	Interval1	-1.16	-0.08	0.98
testosterone	-0.39	0.21	0.86	testosterone	-0.50	0.12	0.75	testosterone	-0.22	0.18	0.60	tesosterone1	-1.94	-0.27	1.50
testis AR	-1.16	-0.44	0.14	testis AR	-0.31	0.22	0.76	testis AR	-0.52	-0.12	0.30	testis AR	-1.27	-0.26	0.82
brain AR	-0.46	0.15	0.78	brain AR	-1.31	-0.61	0.00	brain AR	0.02	0.42	0.84	brain AR	-3.14	-0.40	2.35
Forelimb AR	-2.32	0.16	2.54	Forelimb AR	-0.60	0.45	1.44	Forelimb AR	-1.05	-0.35	0.31	NB: not enough data points for full model			
Corticosterone and glucocorticoid receptor															
Unexposed				Linuron Low				Linuron High				Flutamide			
Percentiles	5.00%	50.00%	95.00%	Percentiles	5.00%	50.00%	95.00%	Percentiles	5.00%	50.00%	95.00%	Not enough data points to fit model			
(Intercept)	-1.00	-0.41	0.18	(Intercept)	-1.00	-0.50	0.01	(Intercept)	-0.29	0.08	0.47				
Egg_no	-1.00	-0.09	0.84	Egg_no	-0.03	0.55	1.18	Egg_no	-0.31	0.08	0.46				
Interval1	-0.68	-0.05	0.55	Interval1	-0.42	0.19	0.86	Interval1	-0.68	-0.26	0.17				
arm_GR1	-0.83	0.13	1.04	arm_GR1	-0.77	-0.14	0.47	arm_GR1	-0.59	-0.14	0.29				
brain_GR1	-0.66	0.13	0.94	brain_GR1	-0.90	-0.33	0.24	brain_GR1	0.25	0.63	1.06				
testis_GR1	-1.15	-0.39	0.30	testis_GR1	-0.26	0.32	0.93	testis_GR1	-0.35	0.08	0.52				
corticosterone1	-0.78	-0.03	0.63	corticosterone1	-0.70	-0.01	0.59	corticosterone1	-0.33	0.15	0.68				

Table S15. Correlations between nuptial pad colour (darker = lower number) and features of morphology and physiology. Model with highest AIC value shown.

<table>
<tr><td colspan="2">Deviance Residuals:</td><td></td><td></td><td></td><td></td></tr>
<tr><td>Min</td><td>1Q</td><td>Median</td><td>3Q</td><td>Max</td><td></td></tr>
<tr><td>-54.589</td><td>-15.87</td><td>-5.382</td><td>18.252</td><td>64.197</td><td></td></tr>
<tr><td></td><td></td><td></td><td></td><td></td><td></td></tr>
<tr><td>Coefficients:</td><td></td><td></td><td></td><td></td><td></td></tr>
<tr><td></td><td>Estimate</td><td>Std. Error</td><td>t</td><td>Value</td><td>Pr(>|t|)</td></tr>
<tr><td>(Intercept)</td><td>-80.303</td><td>101.042</td><td>-0.795</td><td>0.43024</td><td></td></tr>
<tr><td>WGT1</td><td>-5.1506</td><td>3.73258</td><td>-1.38</td><td>0.17331</td><td></td></tr>
<tr><td>SVL1</td><td>4.32945</td><td>2.65518</td><td>1.631</td><td>0.1088</td><td></td></tr>
<tr><td>Interval1</td><td>0.80164</td><td>0.47198</td><td>1.698</td><td>0.09517</td><td>.</td></tr>
<tr><td>testosterone1</td><td>-1.3302</td><td>0.64006</td><td>-2.078</td><td>0.04246</td><td>*</td></tr>
<tr><td>No_spermatogonia1</td><td>-0.1949</td><td>0.06239</td><td>-3.124</td><td>0.00287</td><td>**</td></tr>
<tr><td>no_spermatocytes1</td><td>0.09636</td><td>0.03921</td><td>2.458</td><td>0.01723</td><td>*</td></tr>
<tr><td>spermatozoa1</td><td>1.35643</td><td>0.5333</td><td>2.543</td><td>0.01387</td><td>*</td></tr>
<tr><td></td><td></td><td></td><td></td><td></td><td></td></tr>
<tr><td>AIC:</td><td>599.16</td><td></td><td></td><td></td><td></td></tr>
</table>
